# Engineered Single-Domain Antibodies with High Protease Resistance and Thermal Stability

**DOI:** 10.1371/journal.pone.0028218

**Published:** 2011-11-30

**Authors:** Greg Hussack, Tomoko Hirama, Wen Ding, Roger MacKenzie, Jamshid Tanha

**Affiliations:** 1 Institute for Biological Sciences, National Research Council Canada, Ottawa, Ontario, Canada; 2 Department of Biochemistry, Microbiology and Immunology, Faculty of Medicine, University of Ottawa, Ottawa, Ontario, Canada; 3 School of Environmental Sciences, University of Guelph, Guelph, Ontario, Canada; University of Crete, Greece

## Abstract

The extreme pH and protease-rich environment of the upper gastrointestinal tract is a major obstacle facing orally-administered protein therapeutics, including antibodies. Through protein engineering, several *Clostridium difficile* toxin A-specific heavy chain antibody variable domains (V_H_Hs) were expressed with an additional disulfide bond by introducing Ala/Gly54Cys and Ile78Cys mutations. Mutant antibodies were compared to their wild-type counterparts with respect to expression yield, non-aggregation status, affinity for toxin A, circular dichroism (CD) structural signatures, thermal stability, protease resistance, and toxin A-neutralizing capacity. The mutant V_H_Hs were found to be well expressed, although with lower yields compared to wild-type counterparts, were non-aggregating monomers, retained low nM affinity for toxin A, albeit the majority showed somewhat reduced affinity compared to wild-type counterparts, and were capable of *in vitro* toxin A neutralization in cell-based assays. Far-UV and near-UV CD spectroscopy consistently showed shifts in peak intensity and selective peak minima for wild-type and mutant V_H_H pairs; however, the overall CD profile remained very similar. A significant increase in the thermal unfolding midpoint temperature was observed for all mutants at both neutral and acidic pH. Digestion of the V_H_Hs with the major gastrointestinal proteases, at biologically relevant concentrations, revealed a significant increase in pepsin resistance for all mutants and an increase in chymotrypsin resistance for the majority of mutants. Mutant V_H_H trypsin resistance was similar to that of wild-type V_H_Hs, although the trypsin resistance of one V_H_H mutant was significantly reduced. Therefore, the introduction of a second disulfide bond in the hydrophobic core not only increases V_H_H thermal stability at neutral pH, as previously shown, but also represents a generic strategy to increase V_H_H stability at low pH and impart protease resistance, with only minor perturbations in target binding affinities. These are all desirable characteristics for the design of protein-based oral therapeutics.

## Introduction

The gastrointestinal (GI) tract is the site of numerous microbial infections caused by a range of pathogens, including: *Helicobacter pylori*, *Salmonella* Typhi, *Vibrio cholerae*, *Escherichia coli*, *Campylobacter jejuni*, and *C. difficile*. The current approach for treating most of these infections involves administration of antibiotics, which places selection pressure on the organism, can lead to antibiotic resistance, and suppresses or eliminates beneficial commensal microbes. Disease-causing pathogens of the GI tract rely on a myriad of virulence factors for colonization, adherence, motility, cellular entry, and pathogenesis. These include, but are not limited to: surface-layer proteins, adhesins, invasins, flagella, high-molecular weight toxins, and quorum sensing molecules. Inhibition of bacterial virulence factors that are essential for disease pathogenesis therefore represents a novel, non-antibiotic based strategy to treat infectious diseases, while reducing the risk of microbial resistance and maintaining commensal gut populations [1], [2], [3].

Several approaches are being explored for antivirulence microbial therapy. Inhibition of *E. coli* pilus assembly [4], *Bacillus anthracis* lethal factor [5], [6], Type III secretion systems [7], [8], *Staphylococcus aureus* quorum sensing pathways [9], cholera toxin [10] and *C. difficile* toxins A and B [11], [12], with small molecules and peptides, are examples currently under development. One of the most pursued antivirulence strategies is targeting bacterial toxins with antibodies. Neutralizing antibodies against anthrax [13], shiga toxin [14], cholera toxin [15], botulinum toxin [16] and *C. difficile* toxins [17], [18], [19], [20], [21] have all been successfully isolated and a number of clinical trials involving antibodies to bacterial targets are underway [22]. For human pathogens that secrete toxins into the GI lumen before cellular entry, such as *C. difficile* [23], it may be advantageous to neutralize the toxins within the GI tract. Several studies indicate that oral administration of immunoglobulins (i.e., bovine Ig, human IgA, chicken IgY) may be successful at controlling various GI pathogens, including *C. difficile* [21], [24], rotavirus [25], shigella [26], and enterotoxigenic *E. coli* in humans [27] and neonatal pigs [28]. However, there are major limitations facing orally administered immunotherapeutics, including the susceptibility of antibodies to proteolytic degradation, instability at low pH, high dosing requirements and cost [29].

Recombinant antibody fragments, such as single-domain antibodies (sdAbs) [30], [31] isolated from conventional IgGs (i.e., V_H_s, V_L_s), from the heavy-chain IgG of *Camelidae* species (i.e., V_H_Hs) and from cartilagous shark Ig_NAR_s (i.e., V_NAR_s), are ideal agents to explore for oral immunotherapy [32] because of their small size (12 kDa–15 kDa), high affinity, high protease and thermal stability, high expression, amenability to library selection under denaturing conditions for isolating superstable species and ease of genetic manipulation. Despite possessing relatively high intrinsic protease and pH stability, a limited number of studies have shown that, when administered orally, sdAbs are readily degraded in the low pH pepsin-rich environment of the stomach and by digestive enzymes in the duodenum [33], [34], [35]. Several engineering and selection-based approaches have been undertaken to improve the thermal stability and protease resistance of sdAbs and other recombinant antibody fragments (i.e., scFvs and Fabs). Engineered disulfide bonds [36], [37], [38], [39] and other stabilizing mutations [40] have increased the thermal stability of various recombinant fragments. Library selection of antibodies in the presence of proteases, denaturants, extreme pH, and elevated temperatures has lead to the isolation of antibody fragments with favorable characteristics such as improved thermal and chemical stability, increased protease resistance, and resistance to aggregation [41], [42], [43], [44], [45], [46], [47], [48]. Random mutagenesis approaches have been used to increase the proteolytic stability of V_H_Hs [49]. There has been no universal strategy to increase recombinant antibody thermal and protease stability simultaneously.

In this work, we hypothesized the addition of a non-canonical disulfide bond into the hydrophobic core of llama V_H_Hs between framework region 2 (FR2) and FR3 would not only increase thermal stability at neutral pH, as previously reported [37], [38], [50], but would also impart resistance to proteolytic degradation and increase antibody stability at low pH. To test this hypothesis, we introduced two cysteine residues into a panel of V_H_Hs which neutralize *C. difficile* toxin A (TcdA) [20]. Then, the mutant V_H_Hs were compared to the wild-type V_H_H counterparts with respect to expression yield, tendency for aggregation, antigen binding affinity, CD structural signatures, thermal stability at neutral and acidic pH, susceptibility to GI proteases, and toxin-neutralization capacity.

## Methods

### Chemicals, Reagents, and Cell Lines

All chemicals used in this study were of analytical grade supplied by various companies. Oligonucleotides were synthesized by Operon (Huntsville, AL). The vectors pSJF2H [51] or pMED2 (a modified version of pSJF2H containing *Sfi*I cloning sites) were used for all V_H_H expression in *E. coli* cells (strain TG1) supplied by Stratagene (La Jolla, CA).

### Cloning, Expression, and Purification of V_H_H Mutants

The nomenclature used throughout this work to distinguish between wild-type and mutant V_H_Hs is exemplified as follows: “A4.2” denotes a wild-type V_H_H, “A4.2m” denotes a mutant V_H_H. To construct mutant V_H_Hs with a second disulfide bond, splice-overlap extension-polymerase chain reaction (SOE-PCR) [52] was performed using 4 primers for each V_H_H (Table S1) and two rounds of PCR essentially as described [53]. Ala or Gly and Ile codons at positions 54 and 78 (IMGT numbering system; http://imgt.cines.fr/), respectively, were changed to Cys codons through primer-forced mutation. In the first PCR, two mutagenized overlapping sub-fragments were generated for each V_H_H. The primer pairs used for each V_H_H were as follows: A4.2m (BbsI-VHH and A4.2mR-Cys, A4.2mF-Cys and BamHI-VHH); A5.1m (BbsI-VHH and A5.1mRCys, A4.2mFCys and BamHI-VHH); A19.2m (BbsI-VHH and A19.2mR-Cys, A19.2mF-Cys and BamHI-VHH); A20.1m (A20.1mSfiI-F and A20.1mR-Cys, A20.1mF-Cys and A20.1m*Sfi*I-R); A24.1m (A20.1mSfiI-F and A24.1mR-Cys, A24.1mF-Cys and A20.1mSfiI-R); A26.8m (BbsI-VHH and A26.8mR-Cys, A26.8mF-Cys and BamHI-VHH). Each sub-fragment was gel purified and spliced with its partner fragment in a second PCR. Briefly, 160 ng of each sub-fragment were added to a 50 µL PCR mixture containing *Pfu* DNA polymerase, dNTPs and reaction buffer. The reaction was placed in a thermal cycler and the two fragments were spliced together using a program consisting of a preheating step at 94°C for 5 min and 10 cycles of 94°C for 30 s, 55°C for 30 s, and 72°C for 1 min. To amplify the spliced products, the reaction was heated to 94°C for 3 min, 5 pmol (0.5 µL) of each primer pair was added (BbsI-VHH and BamHI-VHH for A4.2m, A5.1m, A19.2m, and A26.8m; A20.1mSfiI-F and A20.1mSfiI-R for A20.1m and A24.1m), and 35 PCR cycles were performed exactly as described above. The resulting fragments were gel purified, digested with *Bbs*I and *Bam*HI (A4.2m, A5.1m, A19.2m, and A26.8) or *Sfi*I (A20.1m and A24.1m) restriction enzymes, ligated into similarly digested expression vectors (pSJF2H or pMED2), and transformed into TG1 *E. coli* for V_H_H expression. Positive colonies were identified by colony-PCR and DNA sequencing, using the M13RP and M13FP primers (Table S1).

Mutant V_H_Hs were expressed in the same vector as wild-type V_H_Hs [20]. Expression and purification of wild-type and mutant V_H_Hs were performed as described [20], followed by dialysis into phosphate-buffered saline pH 7.3 (PBS), into distilled, deionized water (ddH_2_O) for mass spectrometry (MS) analysis, or into 10 mM sodium phosphate buffer pH 7.3 for CD experiments.

### MS Analysis

Proteolytic peptide fragments of mutant V_H_Hs were created by digestion with cyanogen bromide (CNBr) and trypsin. Briefly, 100 µL reactions containing 50 µg of mutant V_H_H (diluted in PBS), 10 µL of 1 M HCl and 40 µL of CNBr (10 mg/mL stock prepared in 1 M HCl) were digested for 14 h at ambient temperature in the dark. The next day, 100 µL of 1 M Tris-HCl, pH 8.6, and 60 µL of trypsin (100 µg/mL stock; sequencing grade, Roche, Mississauga, ON, Canada) were added directly to the CNBr reaction mixture and incubated for 2 h at 37°C. Samples were then analyzed by non-reducing SDS-PAGE to ensure digestion prior to MS analysis. Nano-flow reversed-phase HPLC MS (nanoRPLC-ESI-MS) with data dependent analysis (DDA) was performed to confirm disulfide bond formation in the mutant V_H_Hs. An aliquot of the CNBr/trypsin digested V_H_Hs was re-suspended in 0.1% formic acid (aq) and analyzed by nanoRPLC-ESI-MS using a nanoAcquity UPLC system coupled to a Q-TOF Ultima™ hybrid quadrupole/TOF mass spectrometer (Waters). The peptides were first loaded onto a 180 µm I.D. ×20 mm 5 µm Symmetry®C18 trap (Waters), then eluted to a 100 µm I.D. ×10 cm 1.7 µm BEH130C18 column (Waters) using a linear gradient from 0% to 36% solvent B (acetonitrile + 0.1% formic acid) in 36 min, 36%–90% solvent B for 2 min. Solvent A was 0.1% formic acid in water. The peptide MS^2^ spectra were searched against mutant V_H_H protein sequences using the Mascot™ database searching algorithm (Matrix Science, London, UK). The MS^2^ spectra of the disulfide-linked peptides were deconvoluted using the MaxEnt 3 program (Waters) for *de novo* sequencing to determine the exact disulfide-linked positions.

### Size Exclusion Chromatography and Affinity Measurements

Mutant V_H_Hs were passed over a Superdex™ 75 (GE Healthcare, Baie-d'Urfé, QC, Canada) size exclusion chromatography column as described [20] to determine their aggregation state. Briefly, V_H_Hs were applied at concentrations ranging from 0.75–1 mg/mL (≅45–60 µM) with a flow rate of 0.5 mL/min in a mobile phase that consisted of HBS-EP running buffer (10 mM HEPES, pH 7.4, 150 mM NaCl, 3 mM EDTA, and 0.005% (v/v) P20 surfactant). The collected fractions from the Superdex™ 75 column were then used directly for surface plasmon resonance (SPR) analysis. All kinetic rate and equilibrium constants were determined as described [20] using a Biacore 3000 instrument (GE Healthcare) and 10,287 resonance units (RUs) of immobilized TcdA. In addition, the dissociation rate constants (*k*_off_s) of mutant V_H_Hs before and after digestion with pepsin were compared by SPR (*see below*).

### CD Spectroscopy

Wild-type and mutant V_H_Hs were analyzed by CD spectroscopy using a Jasco J-815 spectropolarimeter (Jasco, Easton, MD) at pH 7.3 (10 mM sodium phosphate buffer) and at pH 2.0 (10 mM sodium phosphate buffer+50 mM HCl). For all CD experiments performed at pH 2.0, proteins were equilibrated in the above buffer for a minimum of 2 h before scanning. The 50 mM Cl^−^ concentration did contribute to a minor amount of light scatter at wavelengths less than 200 nm. For far-UV CD secondary structure scans and thermal unfolding experiments a 5 mm cuvette containing 1.5 mL of V_H_H at 50 µg/mL (3.2 µM; A_280_≅0.1) was used. V_H_H concentrations of up to 10 µM were initially tested, but signal intensities, expressed in molar ellipticity, were identical to that of 3.2 µM V_H_H concentrations and this concentration also avoided generating compromising signals from protein aggregates formed at high temperatures in thermal unfolding experiments. In these experiments, 4 accumulations were collected for each sample between 190 nm–250 nm with a 1 mm bandwidth, 20 nm/min scan speed and 0.5 nm data pitch. Raw ellipticity data, given in millidegrees (mdeg), was smoothed using the Jasco software, exported, and converted to molar ellipticity, [θ]. To convert from mdeg to molar ellipticity ([θ]) in deg cm^2^/dmol, Equation 1 [54] was used,
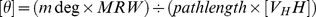
(1)where the mean residue weight, MRW = (molecular weight of the antibody in Da/number of backbone amino acids), pathlength = cell pathlength in mm, and [V_H_H] = concentration of V_H_H in mg/mL. Thermal unfolding was followed at 215 nm with CD measurements taken every 2°C from 30°C to 96°C with a temperature increase of 1°C/min. It should be noted that 0.5°C and 1°C temperature interval measurements, on a select test V_H_H, gave nearly identical *T*_m_ values to 2°C intervals. Molar ellipticity ([θ]) was used to calculate the fraction of protein folded (FF), which is shown in Equation 2 [55],
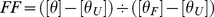
(2)where [θ_F_] and [θ_U_] is the molar ellipticity of the folded (30°C) and unfolded (96°C) states, respectively. The thermal unfolding midpoint temperature (*T*_m_) was obtained by plotting FF against temperature (*T*) and fitting with a sigmoidal Boltzmann function in GraphPad Prism (GraphPad Software, La Jolla, CA). We assumed a temperature of 30°C represented a fully folded V_H_H (FF = 1.0) and a temperature of 96°C represented a fully unfolded V_H_H (FF = 0). In the case of some V_H_Hs with a limited number of lower baseline data points, our *T*_m_ values are minimum estimates. We followed unfolding at 215 nm because of a large difference in ellipticity between folded and unfolded states at this wavelength and because of very low light scattering in samples measured at neutral and acidic pH. A single *T*_m_ replicate for each V_H_H was collected because of the very small standard error in CD-determined *T*_m_ values. For example, a number of previous V_H_H *T*_m_ replicates in our lab, using identical conditions, produced a standard error ranging from ±0.03%–0.63% with an average error of ±0.33%.

To compare the tertiary structures of wild-type and mutant V_H_Hs at neutral and acidic pH, near-UV CD experiments were performed in the range of 250 nm–340 nm using the conditions described above with the exception of a 10 mm cuvette containing 2 mL of protein at 250 µg/mL. In all cases, the ellipticity of buffer blanks were subtracted from experimental values and the reported data is the average of two independent experiments with 4 data accumulations in each.

### Protease Digestion Assays

The sensitivity of wild-type and mutant V_H_Hs to the three major GI proteases pepsin, trypsin, and chymotrypsin was explored. All reactions were performed in 20 µL volumes with 4.8 µg of V_H_H diluted in PBS. For pepsin digestions, reactions contained 17 µL of V_H_H, 2 µL of porcine stomach pepsin (460 U/mg; Sigma, Mississauga, ON, Canada), and 1 µL of 1 M HCl (final pH: 2.0). Final pepsin concentrations in each reaction ranged from 0.1 µg/mL to 100 µg/mL. Digestions were incubated at 37°C for 1 h and neutralized with 1 µL of 1 M NaOH. For trypsin and chymotrypsin digestions, reactions contained 18 µL of V_H_H (diluted in PBS supplemented with 10 mM CaCl_2_) and 2 µL of either trypsin or chymotrypsin (sequencing grade, Roche). Final trypsin/chymotrypsin concentrations ranged from 0.1 µg/mL to 100 µg/mL. Digestions were incubated at 37°C for 1 h and neutralized with 1 µL of protease inhibitor cocktail (Sigma). All neutralized V_H_H-protease reactions and controls (V_H_Hs with no protease) were separated by SDS-PAGE, stained with Coomassie and photographed using an AlphaImager3400 (Alpha Innotech Corporation, San Leandro, CA). To determine the percent of V_H_H retained after protease digestions, densitometry analysis was performed using the AlphaEaseFc software package (Version 7.0.1, Alpha Innotech Corporation) on control and digested V_H_Hs. A total of three independent digestion reactions were performed on all of the V_H_Hs at each protease concentration and replicate digestions were run on separate SDS-PAGE gels. Digestions at the highest protease concentration (100 µg/mL) that were not analyzed by SDS-PAGE were buffer exchanged into ddH_2_O using Millipore Biomax 5K MWCO spin columns (Millipore, Billerica, MA) and subjected to MS analysis to identify the cleavage products, or analyzed by SPR for TcdA binding activity.

### Toxin Neutralization Assay

*In vitro* TcdA neutralization assays were performed essentially as described [20]. Human lung fibroblast cell rounding was reported 24 h post addition of TcdA (100 ng/mL), TcdA+wild-type V_H_H (1000 nM) or TcdA+mutant V_H_H (1000 nM). Specifically, V_H_Hs were added as pooled mixtures of A4.2, A5.1, A20.1, and A26.8 (250 nM each, 1000 nM total) or A4.2m, A5.1m, A20.1m, and A26.8m (250 nM each, 1000 nM total). The percentage of cell rounding was scored visually using light microscopy and the reported values are the average of two independent experiments in which each V_H_H mixture was tested in triplicate.

### Homology Modeling

The SWISS-MODEL online workspace (http://swissmodel.expasy.org/workspace/) [56] was used to construct homology models of A4.2 (wild-type) and A4.2m (mutant) V_H_Hs. The 1qd0A (PDB) V_H_H was used as a template [57], sharing 73.5% and 71.8% homology, respectively. Images of the modeled V_H_Hs were generated using PyMOL (www.pymol.org).

## Results

### Expression and Purification of Mutant V_H_Hs

**Figure 1:**
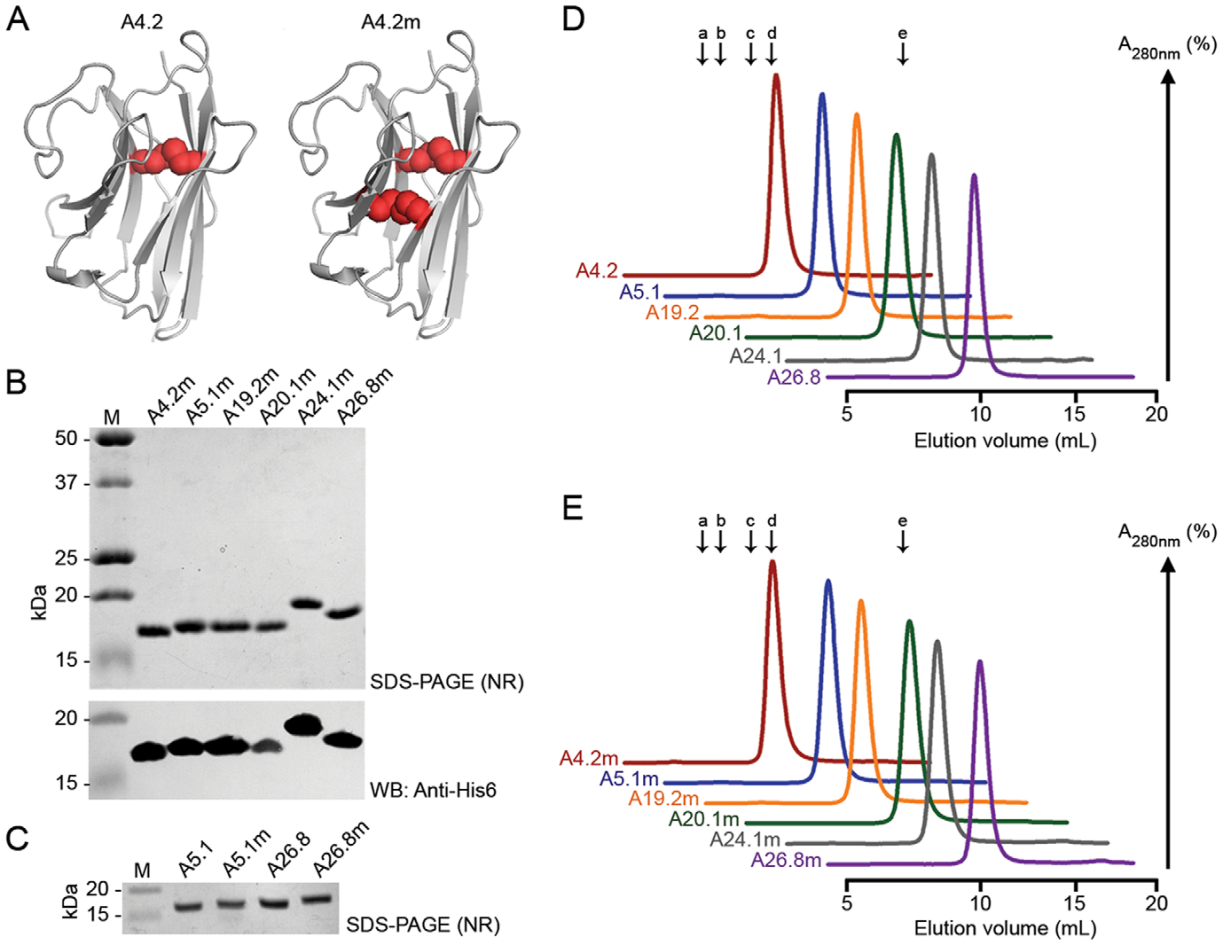
Design, purification, and size exclusion chromatography profiles of disulfide bond mutant V_H_Hs. (**A**) Representative homology models of A4.2 and A4.2m were built on the PDB template 1qd0A V_H_H [57], sharing 73.5% and 71.8% homology, respectively. Disulfide bonds are shown as colored spheres in the hydrophobic core of the V_H_H domains. (**B**) Non-reducing (NR) SDS-PAGE analysis and Western blot (WB) probed with an anti-His_6_ IgG on IMAC-purified mutant V_H_Hs. M: molecular weight marker in kDa. (**C**) Representative SDS-PAGE analysis showing mutant V_H_Hs run slower than the corresponding wild-type V_H_Hs under non-reducing conditions. (**D, E**) Size exclusion chromatography (SEC) analysis of wild-type and mutant V_H_Hs revealed similar size exclusion profiles, indicating the second disulfide bond does not promote the formation of interdomain disulfide-bonds or multimeric mutant V_H_Hs. The elution volumes (V_e_s) of SEC molecular weight standards are shown with arrows and are aligned relative to the A4.2 and A4.2m chromatograms. a: ovalbumin (MW = 43.0 kDa, V_e_ = 8.90 mL); b: carbonic anhydrase (MW = 30.0 kDa, V_e_ = 9.71 mL); c: trypsin inhibitor (MW = 20.1 kDa, V_e_ = 11.06 mL); d: α-lactalbumin (MW = 14.4 kDa, V_e_ = 11.97 mL); e: vitamin B (MW = 1.3 kDa, V_e_ = 18.7 mL). The equation of the line of a standard curve generated from these standards was 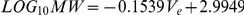 (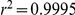). From this equation the V_H_H apparent MWs ranged from 9.8 kDa–13.6 kDa, indicating monomeric V_H_Hs.

Previously, a unique dromedary “V_H_H” was isolated that possessed a naturally occurring disulfide bond between Cys54 and Cys78 residues [58]. When incorporated into several “wild-type” V_H_Hs which possessed only the conserved Cys23/Cys104 disulfide bond, the Cys54/Cys78 disulfide bond increased V_H_H thermal and chemical stabilities [37], [38]. To examine the stabilizing effects of an engineered disulfide bond on llama-derived V_H_Hs, we followed this strategy and chose to introduce two cysteine residues into the hydrophobic core of six *C. difficile* TcdA-specific V_H_Hs [20] by incorporating Ala/Gly54Cys and Ile78Cys point mutations (Fig. 1A, Fig. S1), creating V_H_Hs with two disulfide bonds. Soluble V_H_Hs were extracted from the periplasm of TG1 *E. coli* and purified by immobilized-metal affinity chromatography (IMAC) with purified yields ranging from 3–12 mg/L of bacterial culture. Non-reducing SDS-PAGE and Western blot analysis of the purified products revealed the mutant V_H_Hs were of high purity and did not form interdomain disulfide bonds (Fig. 1B). On non-reducing SDS-PAGE gels, mutant V_H_Hs consistently ran slower than their corresponding wild-type V_H_Hs (Fig. 1C).

### MS Analysis

**Figure 2:**
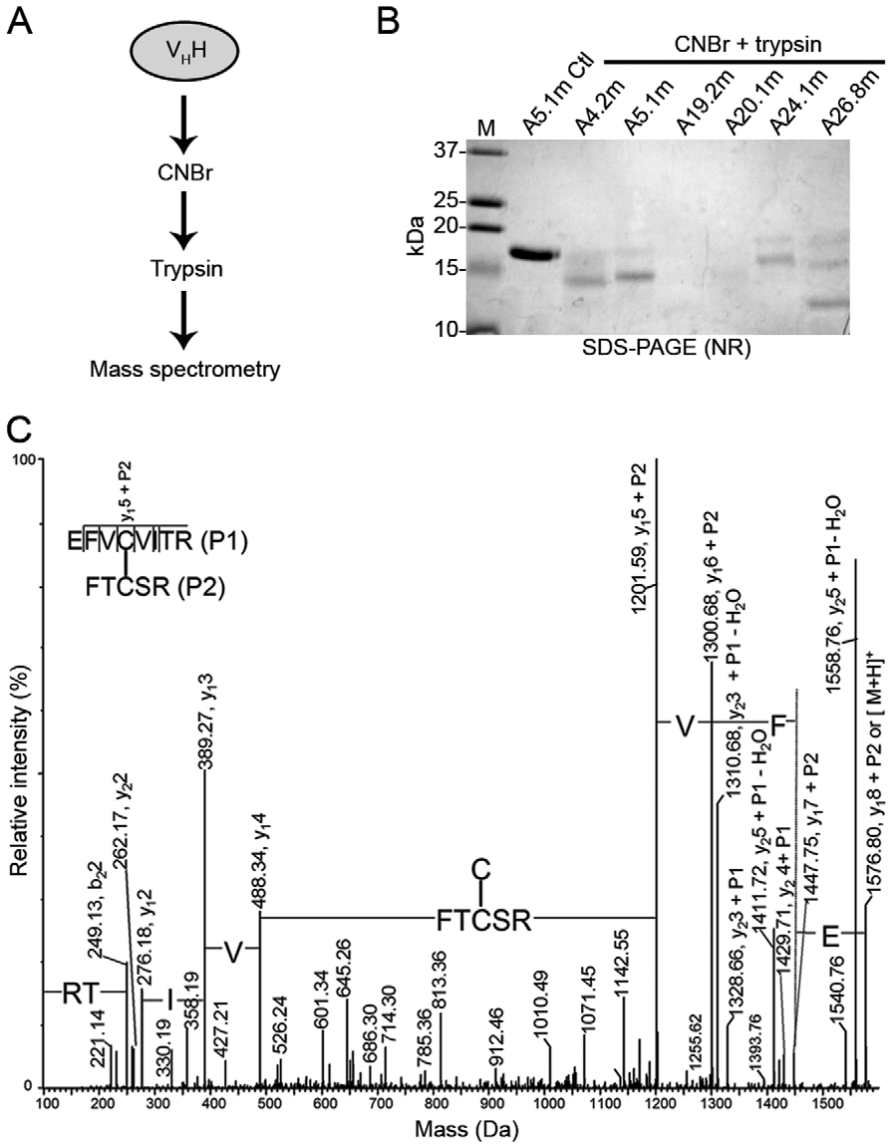
Disulfide bond formation between residues Cys^54^ and Cys^78^ is confirmed by MS^2^. (**A**) Schematic diagram of mutant V_H_H digestion with cyanogen bromide (CNBr) and trypsin before MS^2^ analysis. (**B**) V_H_Hs (3 µg per lane) were subjected to SDS-PAGE analysis under non-reducing (NR) conditions to illustrate near complete digestion with CNBr and trypsin. Untreated A5.1m was added as a control (Ctl). M: molecular weight marker in kDa. (**C**) MaxEnt 3 deconvoluted CID-MS^2^ spectrum of the m/z 526.25 (3+) ion of the disulfide-linked peptide EFVCVITR (P1) – FTCSR (P2), encompassing the Cys^54^–Cys^78^ disulfide bond, from CNBr/trypsin digested A5.1m.

The molecular weights of all mutant V_H_Hs were determined, but were not accurate enough to confirm the formation of the engineered disulfide bond. To precisely confirm the presence of the introduced disulfide bond, mutant V_H_Hs were digested with CNBr and trypsin (Fig. 2A, B) and their digests subjected to MS^2^ analysis. The identification coverage of the mutant V_H_Hs from the analysis of their CNBr/trypsin digests using nanoRPLC-ESI-MS with DDA was more than 30%. The disulfide-linked peptide ions appeared prominent in the survey scan of the DDA experiment when the proteins were digested with a combination of CNBr and trypsin. Peptide fragments linked by the engineered Cys^54^–Cys^78^ disulfide bond (shown in blue text in Fig. S1) were positively identified for all mutant V_H_Hs by manual de-novo sequencing (Table 1). For example, the protein sequence coverage of A5.1m was 43% and a prominent ion at m/z 526.25 (3+) was sequenced as a disulfide-linked peptide EFVCVITR (P1) and FTCSR (P2) as shown (Fig. 2C, Fig. S1, Table 1). An almost complete disulfide-linked y fragment ion series was observed from one peptide with the other peptide attached as a modification via a disulfide bond, which remains intact under collision induced dissociation (CID) [59].

**Table 1:** Disulfide linkage determination of mutant V_H_Hs by MS^2^ analysis.

V_H_H	CNBr/tryptic peptides	MW_for_	MW_exp_	ΔMW
A4.2m	EFV**C**AVSRFT**C**SR	1519.69	1519.70	−0.01
A5.1m	EFV**C**VITRFT**C**SR	1575.75	1575.76	−0.01
A19.2m	EFV**C**GISRFT**C**SR	1519.69	1519.64	0.05
A20.1m	EFV**C**AGSSTGRFT**C**SR	1722.74	1722.84	−0.10
A24.1m	EFV**C**GISWGGGSTRFT**C**SR	2064.91	2064.98	−0.07
A26.8m	EFV**C**VISSTGTSTYYADSVKFT**C**SR	2766.25	2766.33	−0.08

Mutant V_H_Hs were digested with CNBr and trypsin and the peptides analyzed by MS^2^. The peptides containing the Cys^54^–Cys^78^ disulfide linkage are shown with connecting cysteines bolded. A nearly perfect match between MW_for_ and MW_exp_ equates to the presence of the Cys^54^–Cys^78^ disulfide linkage. MW_for_: formula (expected) molecular weight (Da); MW_exp_: experimental molecular weight (Da); 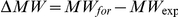.

### Size Exclusion Chromatography and Affinity Measurements

**Figure 3:**
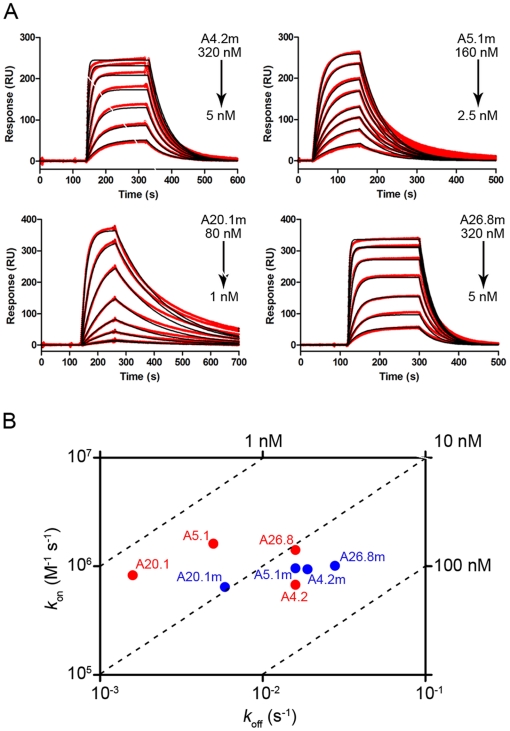
Mutant V_H_Hs retain high affinity binding to TcdA. (**A**) SPR sensorgrams demonstrating mutant V_H_Hs retained high affinity binding to immobilized *C. difficile* TcdA. The range of V_H_H concentrations used in each experiment is shown. Red lines represent measured interaction data, and black lines represent fitted curves. The kinetic and affinity constants are reported in Table 2. Binding of A19.2m and A24.1m to TcdA was non-specific, and the kinetic and affinity constants could not be determined. (**B**) Rate plane plot with iso-affinity diagonals comparing wild-type (red) and mutant V_H_Hs (blue).

Analysis of mutant V_H_Hs on a Superdex™ 75 size exclusion chromatography column produced single, monomeric peaks nearly identical to the profile for wild-type V_H_Hs (Fig. 1D, E), confirming the mutant V_H_Hs are non-aggregating. SPR analysis revealed the specific and high-affinity binding of 4 of 6 mutant V_H_Hs to TcdA (Fig. 3, Table 2). These four were also the strongest TcdA neutralizers. Two mutants (A19.2m and A24.1m) exhibited non-specific binding to reference cell proteins and as a result specific interaction data could not be generated, even at antibody concentrations as high as 3.2 µM. When compared to their wild-type counterparts, the *K*_D_s of 3 TcdA-binding mutants were reduced approximately 2–6 fold (Table 2), while the affinity of one V_H_H was relatively unchanged (*K*_D_s of 24 nM and 20 nM for A4.2 and A4.2m, respectively). The *K*_D_ reductions were largely a result of faster *k*_off_ values and to a much lesser extent influenced by slower *k*_on_ values. In general, these data suggest the Cys^54^–Cys^78^ disulfide bond may slightly distort the V_H_H structure leading to decreases in target binding affinities and decreases in antibody specificity.

**Table 2:** Kinetic and affinity constants of wild-type and mutant V_H_Hs.

V_H_H	Wild-type^a^			Mutant			Fold change in *K*_D_ b
	*k*_on_ (M^−1^ s^−1^)	*k*_off_ (s^−1^)	*K*_D_ (nM)	*k*_on_ (M^−1^ s^−1^)	*k*_off_ (s^−1^)	*K*_D_ (nM)	
A4.2/A4.2m	6.7×10^5^	1.6×10^−2^	24	9.3×10^5^	1.9×10^−2^	20	−1.2
A5.1/A5.1m	1.6×10^6^	5.0×10^−3^	3	9.5×10^5^	1.6×10^−2^	17	+5.7
A19.2/A19.2m	1.4×10^4^	3.9×10^−3^	290	–	–	–	
A20.1/A20.1m	8.2×10^5^	1.6×10^−3^	2	6.4×10^5^	5.9×10^−3^	9.2	+4.6
A24.1/A24.1m	6.0×10^4^	1.6×10^−2^	260	–	–	–	
A26.8/A26.8m	1.4×10^6^	1.6×10^−2^	12	1.0×10^6^	2.8×10^−2^	28	+2.3

a Data obtained from [20].

b Relative to wild-type V_H_H.

### V_H_H Structural and Thermal Stability Characterization

**Figure 4:**
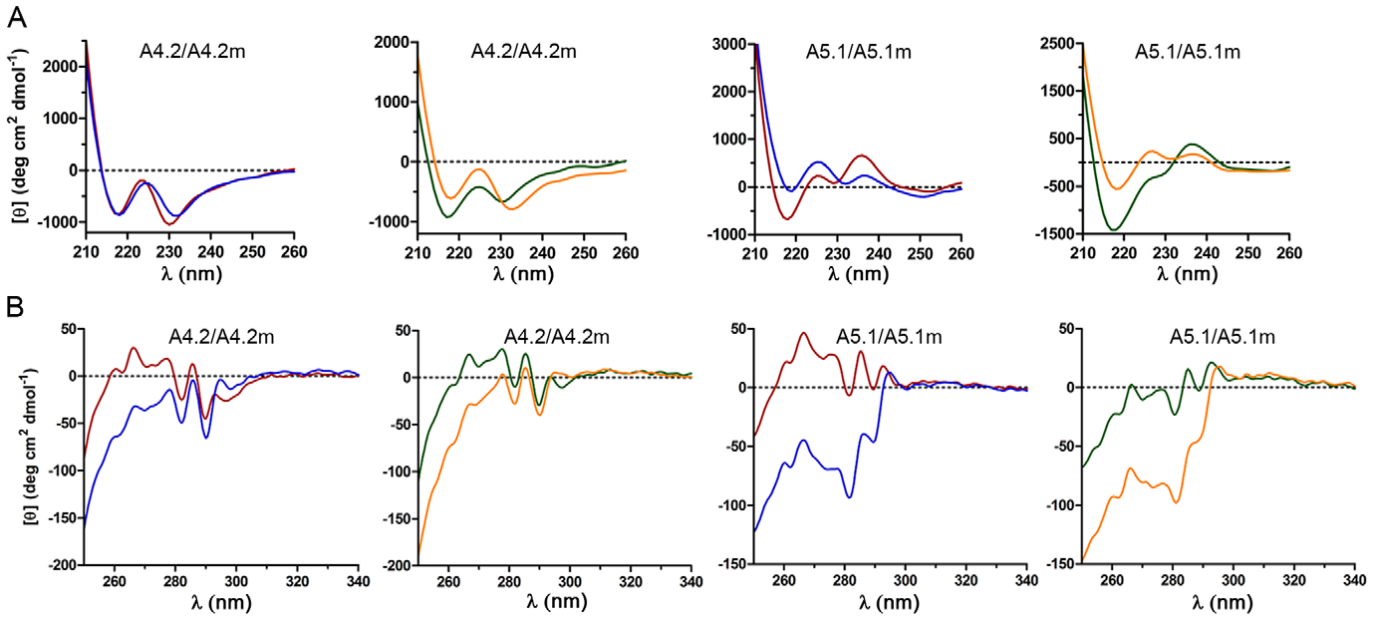
Representative far-UV and near-UV CD spectra of wild-type and mutant V_H_Hs at neutral and acidic pH. Far-UV CD spectra (**A**) and near-UV CD spectra (**B**) of A4.2/A4.2m and A5.1/A5.1m at neutral and acidic pH. Far-UV scans (210 nm–260 nm) were performed at 25°C on V_H_Hs (50 µg/mL) equilibrated for 2 h in 10 mM sodium phosphate buffer (pH 7.3) or 10 mM sodium phosphate buffer+50 mM HCl (pH 2.0) in a 5 mm cuvette. Near-UV scans (250 nm–340 nm) were performed at 25°C on V_H_Hs (250 µg/mL) under similar conditions in a 10 mm cuvette. All spectra represent the mean residue ellipticity from 8 data accumulations collected from 2 independent experiments. Raw data were smoothed using the Jasco software and converted to mean residue ellipticity as described in *Methods*. Red lines: wild-type V_H_H at pH 7.3; blue lines: mutant V_H_H at pH 7.3; green lines: wild-type V_H_H at pH 2.0; orange lines: mutant V_H_H at pH 2.0.

CD experiments were used to examine V_H_H secondary structure, tertiary structure, and thermal stability at both neutral and acidic pH. We first examined V_H_H secondary structure by far-UV CD (Fig. 4A, Fig. S2). Although the overall shape of the far-UV CD spectra from wild-type and mutant V_H_H pairs was similar at a given pH, spectra intensity shifts were observed for all wild-type/mutant pairs. In general, peak minima were seen at 216 nm–218 nm and at 230 nm–235 nm wavelengths but, in almost all cases, the intensity of the peak at 216 nm–218 nm was lower (decreased negative ellipticity) for mutant V_H_Hs. Another prominent feature in the far-UV CD spectra was that mutant V_H_Hs exhibited a near-UV shift in the peak range of 230 nm–235 nm. Wild-type V_H_Hs possessed peak minima around 230 nm–232 nm whereas mutants displayed peak minima in this region around 232 nm–235 nm. Interestingly, A4.2/A4.2m, which of all the wild-type/mutant pairs had the most similar CD spectra at neutral pH, also had the same binding affinity for TcdA.

We next examined V_H_H tertiary structures with near-UV CD spectroscopy (Fig. 4B, Fig. S3). The CD spectra in this region (250 nm–320 nm) come primarily from aromatic residues within the V_H_H, with Phe contributing in the range of 250 nm–270 nm, Tyr contributing in the range of 270 nm–290 nm, and Trp contributing in the range of 280 nm–300 nm. Overall, the near-UV spectra profiles were similar between wild-type and mutant V_H_H pairs. Spectra from wild-type and mutant pairs shared nearly identical peak wavelengths; however, between 250 nm to 295 nm, the ellipticity of mutant V_H_Hs was consistently more negative than wild-type V_H_Hs. There were also subtle differences in peaks occurring around 297 nm, with mutant V_H_Hs exhibiting a minor but consistent shift to the right. Three of the four wild-type/mutant pairs (A4.2/A4.2m, A5.1/A5.1m, and A20.1m/A20.1m) produced predominantly negative ellipticity, whereas the A26.8/A26.8m pair remained positive. The contributions of the second disulfide bond cannot be ruled out as a factor which may augment the contribution of aromatic residues to ellipticity (increasing negatively) of the mutants.

**Figure 5:**
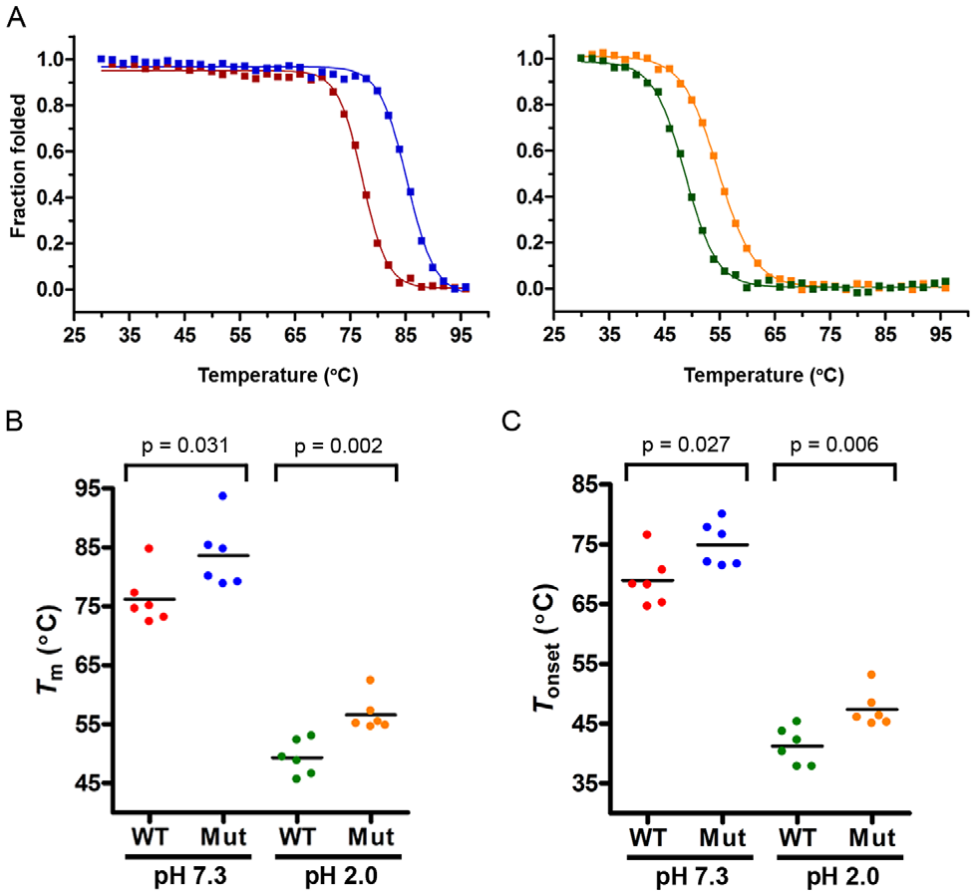
Mutant V_H_H thermal unfolding midpoint temperatures are significantly greater than those of wild-type V_H_Hs. (**A**) Representative example showing the thermal unfolding of A26.8 (WT) and A26.8m (Mut) at neutral pH (*left*) and acidic pH (*right*). V_H_H thermal unfolding midpoint temperatures (*T*_m_s) were determined using CD spectroscopy by following antibody unfolding (50 µg/mL) at 215 nm in 10 mM sodium phosphate buffer +/−50 mM HCl. Raw data were converted to fraction folded, as described in *Methods*, and the *T*_m_ was determined by Boltzmann sigmoidal curve fitting (r^2^ ranging from 0.9965–0.9995). *T*_onset_ was determined from the same curve and was defined as the temperature at which 5% of the V_H_H was unfolded. Red lines: wild-type V_H_H at pH 7.3; blue lines: mutant V_H_H at pH 7.3; green lines: wild-type V_H_H at pH 2.0; orange lines: mutant V_H_H at pH 2.0. (**B**) Summary of V_H_H *T*_m_s. (**C**) Summary of V_H_H *T*_onset_s. In *B* and *C*, dots represent individual V_H_Hs and the black bars represent the mean *T*_m_ or *T*_onset_, respectively. P-values were determined using the unpaired two-tailed *t*-test.

Finally, temperature-induced unfolding experiments were conducted in order to determine V_H_H *T*_m_s and *T*_onset_s by following changes in V_H_H ellipticity at 215 nm (Fig. 5, Fig. S4, Table 3, Table S2). All V_H_Hs exhibited sigmoidal melting curves, indicative of cooperative unfolding of a protein that exists in either a folded or unfolded state. The wild-type V_H_Hs already have high *T*_m_s (as high as 84.7°C) – significantly higher than those reported for other V_H_Hs [60]. At neutral pH, all mutant V_H_Hs had significantly higher thermal unfolding midpoint temperatures (p = 0.031, unpaired two-tailed *t*-test) than their wild-type V_H_H counterparts. The *T*_m_ values of mutants ranged from 78.8°C to 93.6°C, with one mutant, A5.1m, having a *T*_m_ 11.6°C higher than wild-type (A5.1). The increase in mutant V_H_H *T*_m_s relative to wild-type ranged from 3.7°C to 11.6°C. Overall, at neutral pH, the mean *T*_m_ ± SEM was 76.2°C±1.8°C and 83.6°C±2.3°C for wild-type and mutant V_H_Hs, respectively (Fig. 5B). These findings are in agreement with previous reports that showed significant increases in the *T*_m_s of disulfide bond engineered V_H_Hs [37], [38], [50]. In a second series of experiments, temperature-induced unfolding was conducted at pH 2.0 by once again following V_H_H ellipticity changes at 215 nm (Fig. 5, Fig. S4, Table 3). At acidic pH a considerable reduction in *T*_m_ was observed for both wild-type (22.1°C to 32.4°C) and mutant V_H_Hs (23.7°C to 31.2°C) when compared to the *T*_m_ values recorded at pH 7.3. However, at acidic pH the *T*_m_ of all six mutants was still significantly higher than the corresponding wild-type V_H_Hs (p = 0.002, unpaired two-tailed *t*-test). In acid, the increase in mutant V_H_H *T*_m_s relative to wild-type ranged from 2.1°C to 11.6°C, which is a nearly identical spread in temperature increases to that seen at neutral pH. Overall, at pH 2.0, the mean *T*_m_ ± SEM was 49.3°C±1.2°C and 56.6°C±1.2°C for wild-type and mutant V_H_Hs, respectively (Fig. 5B). Interestingly, the highest *T*_m_ gains at both pHs were seen for the four strongest neutralizers. The *T*_m_ differences between wild-type/mutant pairs are more significant at acidic pH than neutral pH. Taken together, these results (Table 3; Fig. 5) suggest the Cys^54^–Cys^78^ disulfide bond may stabilize the V_H_Hs from acid-induced denaturation. Using our thermal unfolding curves, we also identified V_H_H *T*_onset_ temperatures, the temperature at which 5% of the V_H_H was unfolded (Fig. 5C; Table S2). The *T*_onset_ of mutant V_H_Hs was significantly higher than wild-type V_H_Hs at both neutral and acidic pH (p = 0.027 and p = 0.006, respectively, unpaired two-tailed *t*-test). The *T*_onset_ differences between wild-type/mutant pairs are more significant at acidic pH than neutral pH. At pH 7.3, the mean *T*_onset_ ± SEM was 68.9°C±1.8°C and 74.9°C±1.5°C for wild-type and mutant V_H_Hs, respectively. At pH 2.0, the mean *T*_onset_ ± SEM was 41.2°C±1.3°C and 47.3°C±1.3°C for wild-type and mutant V_H_Hs, respectively. Therefore, the lowest *T*_onset_ for the mutants was 45.0°C, whereas two of the wild-type V_H_Hs (A5.1, A20.1) already had *T*_onset_s of ∼37°C at pH 2.0 (physiological stomach conditions).

**Table 3:** Thermal unfolding midpoint temperatures (*T*_m_) of wild-type and mutant V_H_Hs.

V_H_H	*T*_m_ (°C) at pH 7.3			*T*_m_ (°C) at pH 2.0		
	Wild-type	Mutant	Δ*T*_m_	Wild-type	Mutant	Δ*T*_m_
A4.2/A4.2m	84.7*	93.6*	8.9	52.3	62.4	10.1
A5.1/A5.1m	73.1	84.7*	11.6	45.6	57.2	11.6
A19.2/A19.2m	75.1	78.8	3.7	53.0	55.1	2.1
A20.1/A20.1m	72.4	79.1	6.7	46.6	55.4	8.8
A24.1/A24.1m	74.6	80.1	5.5	49.4	54.6	5.2
A26.8/A26.8m	77.2	85.3*	8.1	48.8	54.8	6.0

*Minimum estimated *T*_m_.

### Protease Digestion Assays

**Figure 6:**
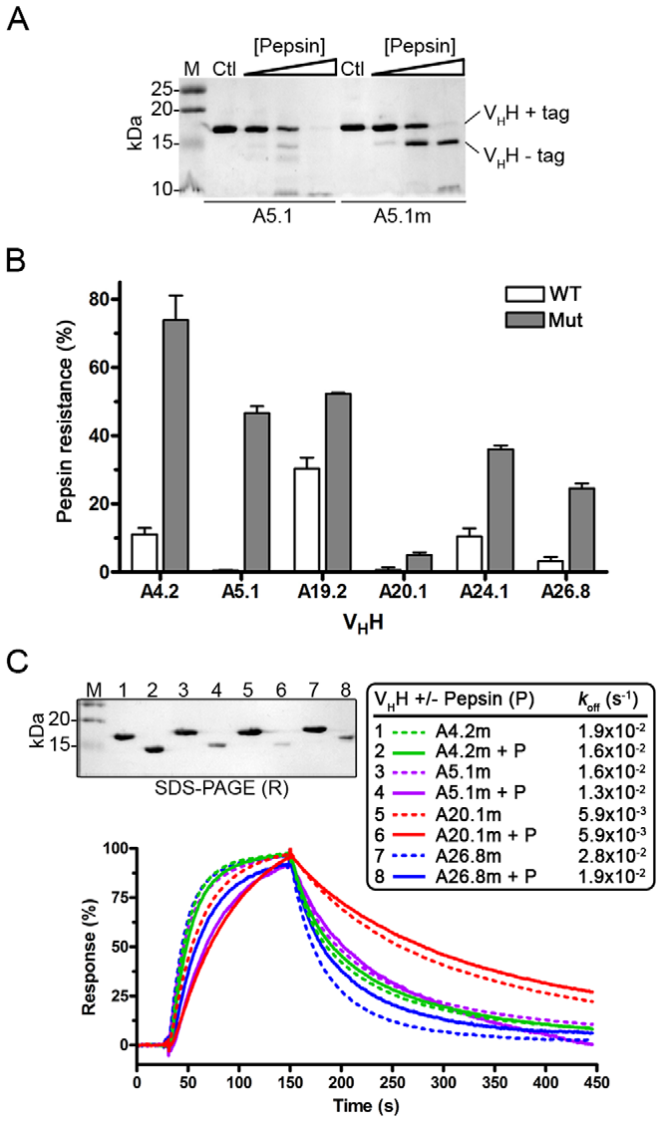
Mutant V_H_Hs are resistant to pepsin degradation. (**A**) Representative SDS-PAGE analysis showing the separation of A5.1 and A5.1m V_H_Hs after digestion with various concentrations of pepsin (increasing from left to right: 1 µg/mL, 10 µg/mL and 100 µg/mL) at pH 2.0 and 37°C for 1 h. Control V_H_Hs (Ctl) were incubated under the same conditions without pepsin. Three micrograms of protein was loaded per lane. Bands appearing ∼2 kDa below the full-length V_H_H (“V_H_H+tag”) were identified by MS (data not shown) as V_H_Hs cleaved within the C-terminal c-Myc tag (“V_H_H−tag”), as shown before with protease-digested human V_H_s [61]. (**B**) Summary of V_H_H resistance profiles to 100 µg/mL pepsin treatment. Resistance values were obtained by densitometric measurements of pepsin-treated V_H_Hs relative to controls (as in Fig. 6A). Error bars represent the SEM obtained from 3 independent digestions for each V_H_H. (**C**) SPR analysis (*bottom*) on mutant V_H_Hs digested with pepsin (100 µg/mL, 1 h, 37°C). The pepsin-treated V_H_Hs retained their ability to bind surface-immobilized TcdA. SDS-PAGE (*top*) showing untreated (lanes 1, 3, 5, 7) and pepsin-digested (lanes 2, 4, 6, 8) V_H_Hs used for SPR. The contents of lanes 1 thru 8 are described in the box in *C*. Normalized *k*_off_s for pepsin treated V_H_Hs were similar to the *k*_off_ of untreated controls (*box* and Table 2). M: molecular weight markers in kDa; WT: wild-type V_H_H; Mut: mutant V_H_H; P: pepsin; R: reducing SDS-PAGE conditions.

Proteins traveling through the GI tract encounter low pH and digestive enzymes in the stomach. We therefore asked if the Cys^54^–Cys^78^ disulfide bond improved V_H_H resistance to proteolytic degradation. We compared the effects of the major GI proteases pepsin, trypsin, and chymotrypsin on wild-type and mutant V_H_Hs through SDS-PAGE and MS analysis. Initially, protease concentrations of 0.1 µg/mL, 1 µg/mL, 10 µg/mL, and 100 µg/mL were explored. When the lowest concentrations of proteases (0.1 µg/mL and 1 µg/mL) were used in digestion reactions, wild-type and mutants appeared similar to undigested controls on SDS-PAGE (data not shown). Similarly, V_H_Hs were only moderately susceptible to protease degradation at 10 µg/mL (data not shown). In order to see clear differences in the proteolytic susceptibility of wild-type and mutant V_H_Hs, all remaining digestions were performed at protease concentrations of 100 µg/mL. SDS-PAGE analysis of pepsin-digested wild-type and mutant V_H_Hs showed a reduction in V_H_H size from ∼16 kDa (control) to either ∼14 kDa, or complete digestion to smaller fragments (Fig. 6A). The band at ∼14 kDa routinely appeared in digestions with each of the proteases. Similar to V_H_ protease digestion studies [61], MS mass analysis on the ∼14 kDa products revealed cleavage at various positions within the V_H_H C-terminal c-Myc epitope tag. Loss of the epitope tag corresponded to reductions of 1641.7 Da, 1754.8 Da, and 1641.7 Da for pepsin, trypsin, and chymotrypsin digested V_H_Hs, respectively (data not shown).

**Figure 7:**
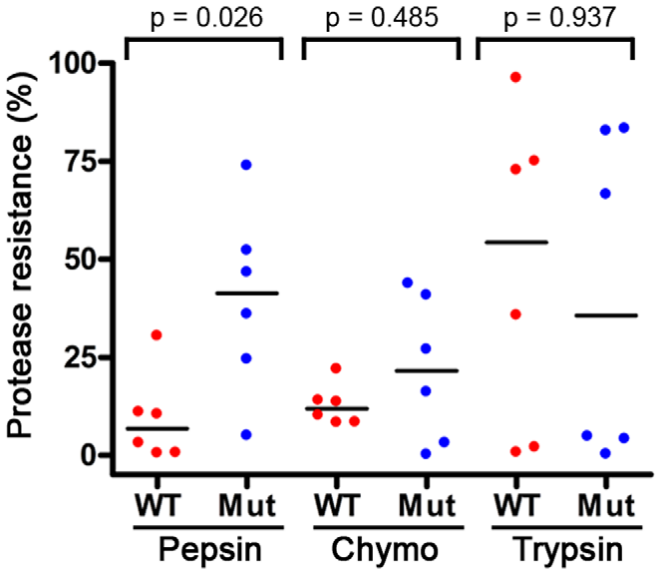
Summary of V_H_H resistance profiles to pepsin, trypsin, and chymotrypsin. V_H_H resistance to the major GI proteases was determined by proteolytic digestion (100 µg/mL protease, 37°C, 1 h) and SDS-PAGE densitometry analysis. Dots represent the mean (n = 3) protease resistance profile of each V_H_H relative to undigested controls and the black bars represent the median resistance of each group. P-values were determined using the unpaired two-tailed Mann-Whitney *U* test. WT: wild-type V_H_H; Mut: mutant V_H_H; Chymo: chymotrypsin.

Overall, significant increases in pepsin resistance were found for all mutant V_H_Hs compared to their wild-type counterparts (p = 0.026, Mann-Whitney *U* test) (Fig. 6B; Fig. 7; Table 4). The increase in mutant V_H_H pepsin resistance relative to corresponding wild-type ranged from almost 4.5% to 63% (Table 4). For example, A5.1 was completely degraded after incubation with pepsin, while nearly 50% of A5.1m remained intact (Fig. 6A, B). The biggest increase in pepsin resistance was found for A4.2m, where an almost 63% increase in intact V_H_H structure was found relative to A4.2. Interestingly, A4.2m also had the highest *T*_m_ and *T*_onset_ at pH 2.0 (Table 3; Table S2), the same pH at which the pepsin digestions were performed. Increases in mutant V_H_H resistance to chymotrypsin were not as universal (Fig. 7; Fig. S5, Table 4) but, nonetheless, 4 of 6 mutant V_H_Hs showed increased resistance to chymotrypsin, with significant increases found in clones A5.1m, A24.1m, and A26.8m (p<0.05) compared to their wild-type counterparts. No statistical differences were found between trypsin digested wild-type and mutant V_H_Hs (Fig. 7; Fig. S5, Table 4), except for A4.2m, where trypsin resistance was actually reduced from almost 36% in the wild-type V_H_H to almost 5% in the mutant. Both the wild-type and mutant versions of A19.2 and A26.8 were very susceptible to trypsin degradation.

**Table 4:** Protease resistance profiles of wild-type and mutant V_H_Hs to the major GI proteases.

V_H_H	Pepsin resistance (%)		Chymotrypsin resistance (%)		Trypsin resistance (%)	
	Wild-type	Mutant	Wild-type	Mutant	Wild-type	Mutant
A4.2/A4.2m	11.08±1.88	73.87±7.23	13.60±6.50	3.18±1.10	35.72±7.08	4.80±0.61
A5.1/A5.1m	0.53±0.15	46.63±1.99	14.03±3.15	27.00±4.05	96.23±7.09	83.30±4.96
A19.2/A19.2m	30.37±3.16	52.27±0.32	8.30±1.14	0.18±0.10	0.73±0.73	0.27±0.27
A20.1/A20.1m	0.68±0.68	5.04±0.76	10.17±1.85	16.17±5.26	72.77±4.85	82.80±1.97
A24.1/A24.1m	10.45±2.39	36.02±1.11	22.03±5.01	43.80±2.08	75.03±9.63	66.50±3.58
A26.8/A26.8m	3.17±1.24	24.56±1.45	8.40±1.23	40.83±8.81	2.03±2.03	4.10±1.27

All V_H_H digestions were performed at 37°C for 1 h in the presence of 100 µg/mL protease. Resistance values were obtained by comparing the intensity of protease-digested V_H_Hs relative to untreated controls using SDS-PAGE and imaging software. See Fig. 6A as an example. Values represent the mean ± SEM (n = 3). Data were incorporated into Fig. 6B and Fig. S5.

**Figure 8:**
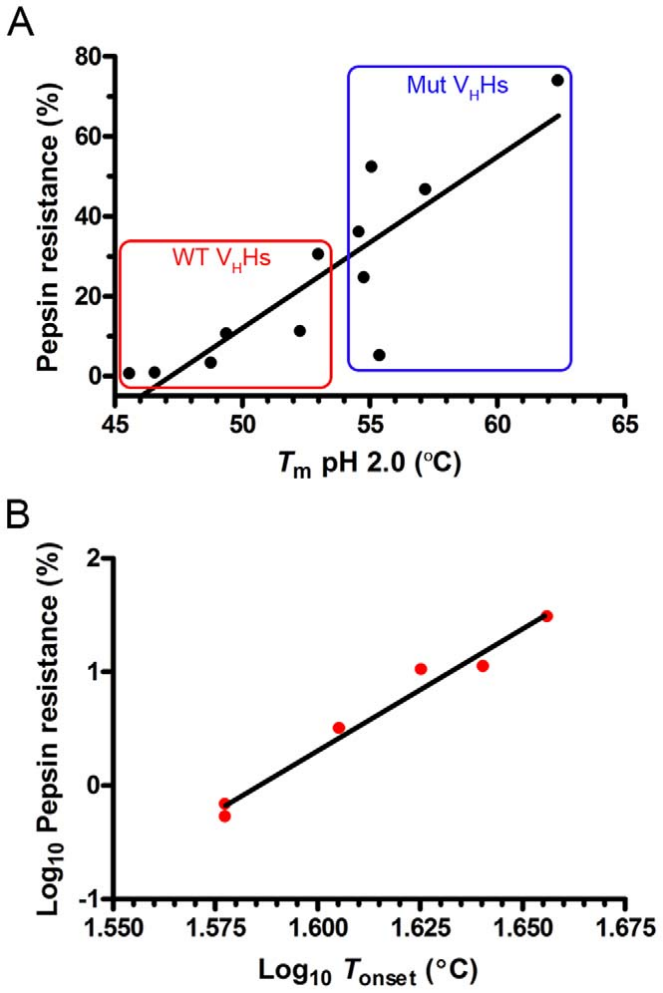
Correlation between V_H_H pepsin resistance and thermal stability at acidic pH. (**A**) Linear regression between V_H_H pepsin resistance and V_H_H *T*_m_ at pH 2.0. Red and blue boxes show the wild-type (WT) and mutant (Mut) V_H_Hs, respectively. Linear regression analysis gave a correlation coefficient of r^2^ = 0.735 and a significantly non-zero slope of the line (p = 0.0004). (**B**) Linear regression between wild-type V_H_H pepsin resistance and wild-type V_H_H *T*_onset_ at pH 2.0. The *T*_onset_ is defined as the temperature at which 5% of the V_H_H is unfolded. Linear regression analysis gave a correlation coefficient of r^2^ = 0.975 and a significantly non-zero slope of the line (p = 0.0002).

A correlation was observed between V_H_H pepsin resistance and *T*_m_s at pH 2.0 (r^2^ = 0.735, Fig. 8A). The wild-type V_H_Hs with lower *T*_m_s occupied the low protease resistance region of the graph, the mutants with higher *T*_m_s occupied the high protease resistance region of the graph. There was also a moderate correlation between V_H_H pepsin resistance and *T*_m_s at pH 7.3 (r^2^ = 0.500, data not shown). No correlation was evident between V_H_H trypsin resistance and *T*_m_s at pH 7.3 or pH 2.0 (r^2^ = 0.138 and r^2^ = 0.138, respectively) or between V_H_H chymotrypsin resistance and *T*_m_s at pH 7.3 or pH 2.0 (r^2^ = 0.012 and r^2^ = 0.004, respectively). In addition, a strong correlation between wild-type V_H_H pepsin resistance and wild-type V_H_H *T*_onset_ at pH 2.0 was noted (r^2^ = 0.975, Fig. 8B, Table S2). No correlation was evident between mutant V_H_H pepsin resistance and mutant V_H_H *T*_onset_ at pH 2.0 (r^2^ = 0.191), presumably because mutant V_H_H *T*_onset_ temperatures were much higher than the temperature at which pepsin digestions were performed (37°C). Interestingly, we also noted a correlation between V_H_H trypsin resistance and the theoretical number of trypsin cleavage sites located within the whole V_H_H (r^2^ = 0.822) or located within the V_H_H CDR (r^2^ = 0.681) regions (Table S3, Fig. S6). No correlation was found between V_H_H pepsin or chymotrypsin resistance and the theoretical number of pepsin or chymotrypsin cleavage sites, respectively (Fig. S6).

The ability of pepsin-treated mutants (A4.2m, A5.1m, A20.1m, and A26.8m) to bind TcdA was evaluated by SPR. SPR analyses confirmed the mutants (“V_H_H−tag”; *see* Fig. 6A) retained TcdA binding as their *k*_off_ values were essentially the same as those of untreated controls (Table 2; Fig. 6C). SPR analysis on pepsin-digested wild-type V_H_Hs could not be performed since these V_H_Hs were significantly degraded by pepsin. These experiments highlight the profound impact a second disulfide bond in the hydrophobic core has on V_H_H conformational stability at low pH and resistance to proteolytic degradation by pepsin.

### Toxin Neutralization Assay

**Figure 9:**
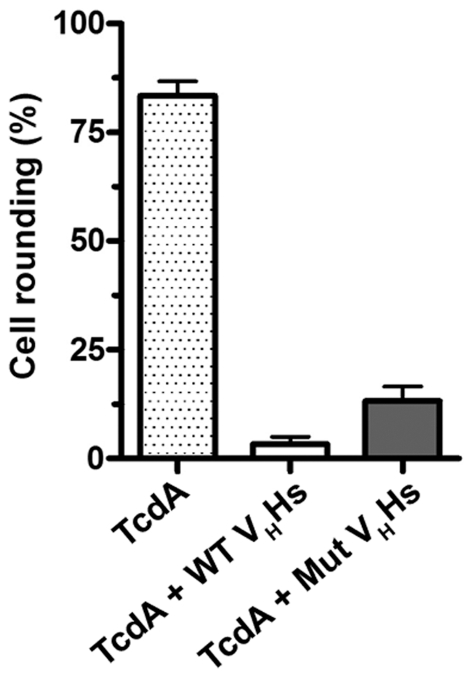
Mutant V_H_Hs retain TcdA-neutralizing capacity. Confluent monolayers of IMR-90 human lung fibroblasts were incubated with TcdA (100 ng/mL) or TcdA+V_H_Hs (1000 nM) for 24 h, and the percentage of cells rounded was scored using a light microscope from 0% to 100%. V_H_Hs (wild-type (WT) or mutant (Mut)) were added as pooled mixtures of A4.2, A5.1, A20.1, and A26.8 (250 nM each) or A4.2m, A5.1m, A20.1m, and A26.8m (250 nM each).

Mutant V_H_Hs retained their ability to neutralize to cytotoxic effects of TcdA on monolayers of fibroblast cells. Comparison of the neutralization capacity of pooled mixtures (1000 nM total) of wild-type and mutant V_H_Hs revealed mutants performed nearly as well as wild-types at reducing TcdA-mediated cell rounding (Fig. 9). Given that 3 of 4 mutants showed weaker affinity for TcdA the reduction in neutralizing capacity relative to wild-type V_H_Hs was not unexpected.

## Discussion

The rapid development of bacterial resistance to most major classes of antibiotics has created a demand for novel therapeutics in the fight against infectious diseases. One of the most pursued non-antibiotic strategies involves targeting bacterial virulence factors with small molecules and antibodies. For some pathogens, inhibition of toxins and colonization factors within the GI tract may be an effective means of disease control. Oral immunotherapy for treating infectious diseases has had limited success due to the instability of immunoglobulins in the extreme pH and protease-rich environment of the GI tract. Here, through protein engineering, we increased the protease, acid and thermal stability of llama-derived sdAbs (V_H_Hs) which target and neutralize *C. difficile* toxin A without dramatically affecting biological function.

Our stabilization strategy involved the substitutions of two amino acid residues at positions 54 and 78 for cysteine, allowing for the formation of a second, non-native disulfide bond between FR2 and FR3 in the V_H_H hydrophobic core. Incorporation of a disulfide bond at these positions has been previously reported in camelid V_H_Hs [37], [38], [50] and was found to increase V_H_H chemical and thermal stability. We hypothesized that the additional disulfide bond may also enhance V_H_H resistance to proteases, especially in denaturing acidic conditions.

To test this hypothesis, we generated the disulfide bond mutants and compared them to their wild-type counterparts containing only the native disulfide bond between residues 23 and 104. Mutant V_H_Hs were well expressed in *E. coli* when targeted to the periplasmic space, although with lower yields compared to wild-type V_H_H counterparts, and all were non-aggregating monomers as determined by size exclusion chromatography. To confirm disulfide bond formation, we used a combination of proteolytic and chemical digestion coupled with MS^2^ to precisely identify V_H_H peptide fragments harboring the introduced disulfide bond. This approach is preferred over the Ellman's assay approach for the determination of disulfide linkage formation, as it requires less quantities of protein and reveals the positional identity of Cys pairs in a given disulfide bond. The latter information is important, as there is also the possibility that the two engineered Cys residues, besides forming the desired disulfide bond may form undesired disulfide bonds with the two conserved Cys residues at positions 23 and 104. After confirming disulfide bond formation in our mutants, SPR binding experiments revealed most mutant V_H_Hs possessed 1- to 5-fold weaker affinity constants relative to wild-type, which is consistent with observations by others of up to 3-fold reductions in the affinities of V_H_Hs containing the same introduced disulfide bond [38], [50]. However, for the two weak neutralizing V_H_Hs, A19.2m and A24.1m, the non-canonical disulfide linkage compromised specificity.

We used CD spectroscopy to compare wild-type and mutant V_H_H secondary structure, tertiary structure and thermal stability (*T*_m_ and *T*_onset_). Comparisons of V_H_H secondary and tertiary structure with far-UV and near-UV CD spectroscopy strongly suggested structural differences between wild-type and mutants, at both neutral and acidic pH. For all mutants, peak intensity and selective peak minima shifts were observed, although the overall spectral profiles remained very similar in all wild-type/mutant pairs. More specifically, mutants consistently showed rightward peak shifts in the peak range of 230 nm–235 nm (far-UV CD spectra) and around 297 nm (near-UV CD spectra) compared to wild-type V_H_Hs. Such patterns may be used as signatures that could be used to quickly identify V_H_Hs containing a properly formed non-canonical disulfide bond, as could SDS-PAGE motility values since, compared to wild-type V_H_Hs, mutants consistently moved slower in SDS-PAGE gels. Thus, the far- and near-UV CD spectral data suggests the introduced disulfide bond changes the structure of V_H_Hs. This is consistent with the observed perturbations in affinities and specificities and increased GI protease resistance of the mutant V_H_Hs compared to the wild-types (*see below*). We used CD spectroscopy thermal denaturation experiments to show a profound and significant increase in the *T*_m_s and *T*_onset_s of mutant V_H_Hs at both neutral and acidic pH. These mutants are more thermostable than previously reported V_H_s, which were affinity selected from a V_H_ phage display library under stability pressure [45]. The beneficial effect of the non-canonical disulfide linkage on *T*_m_s varies widely, with *T*_m_ increases ranging from ≈4°C to ≈12°C. This suggests that for the mutant V_H_Hs with a higher thermostability gain, the non-canonical disulfide linkage may have been a better fit to the overall fold. A19.2m and A24.1m showed the lowest thermostability gains and, if it is true that this is because of an unfit disulfide linkage, it would explain why they were transformed into non-specific binders upon mutation. For A4.2m on the other hand, the non-canonical disulfide linkage seems to be a natural fit, as it increased its *T*_m_ the most (by almost 12°C) and significantly improved GI protease resistance (with the highest increase in pepsin resistance; *see below*), all without adversely affecting the *K*_D_. We also observed a correlation between pepsin resistance and *T*_m_, and this has implications in terms of using heat as the selective pressure for selecting pepsin resistant antibody fragments by *in vitro* evolutionary approaches.

Most likely, mutants (exhibiting higher *T*_m_s) also have higher thermodynamic stability since thermodynamic stability generally increases with *T*_m_ [62]. This has been shown to be the case for both V_H_ and V_H_H domains as well [38], [45]. In the instance of V_H_Hs, it has been shown that the introduction of the Cys54/Cys78 disulfide linkage used in our study into V_H_Hs led to increases in both *T*_m_ and thermodynamic stability. Proteins with higher *T*_m_ are also less likely to unfold [62]. These may be the reasons why our mutants were more resistant to acid-induced unfolding at 37°C, supported by the higher *T*_onset_s and pepsin resistance of our mutant V_H_Hs (*see below*). Consistent with this, in a previous study, human V_H_s which were more resistant to acid-induced aggregation, a phenomenon encouraged/initiated by protein unfolding, had higher *T*_m_s and thermodynamic stabilities [45]. The improved reversibility of thermal unfolding of mutant V_H_Hs compared to their wild-type counterparts under acidic conditions in our work (data not shown) indicates that the introduced disulfide linkage may also render V_H_Hs with aggregation resistant unfolded states [48], in addition to higher thermodynamic stability. Hagihara et al [37] showed that the introduction of the same Cys54/Cys78 disulfide linkage into a V_H_H, in addition to increasing its *T*_m_, led to decreases in its enthalpy and entropy changes of unfolding. The enthalpy and entropy measurements indicated that the stabilization effect of the extra disulfide linkage in V_H_Hs may be related to factors such as loop entropy, internal interactions such as hydrogen bonding and van der Waals interactions and hydration of the native and unfolded states.

We also examined the resistance profiles of the disulfide bond mutants to the major GI proteases. Mutant V_H_Hs were universally more resistant to pepsin and many were more resistance to chymotrypsin when compared to their wild-type counterparts. Protease sensitivity is a function of many variables including the location of proteolytic sites (e.g., loops *vs* protein core in antibodies), the theoretical number of proteolytic sites, and protein compactness and thermodynamic stability [63], [64]. Since each wild-type and mutant V_H_H pair possessed the identical number of theoretical protease cleavage sites, we speculate that the second disulfide bond presents a more compact and thermodynamically stable V_H_H structure, preventing pepsin and chymotrypsin from accessing proteolytic cleavage sites. This view is consistent with the increased *T*_m_s in mutants (an indicator of mutants' increased thermostability), the positive correlation between pepsin resistance and *T*_m_ (Fig. 8), and the lack of correlation between pepsin/chymotrypsin resistance and the number of theoretical protease cleavage sites (Fig. S6). The pepsin resistance *vs T*_m_/*T*_onset_ correlation curves also point to the fact that structural compactness and thermodynamic stability plays a more prominent role in pepsin resistance, which is understandable given that pepsin requires protein unfolding for efficient digestion. This benefit is not realized for mutants against trypsin, possibly because their cleavage sites are at hydrophilic residues (Lys or Arg) which must be in more exposed regions of the V_H_H, possibly located in the CDR regions. Further, these regions would not be protected by stabilizing the core of the structure. The positive correlation between V_H_H trypsin resistance and the number of theoretical trypsin cleavage sites is a testament to this (Fig. S6). Harmsen et al [49] have suggested the CDR regions of V_H_Hs to be the most sensitive sites to proteolysis due to their flexibility and exposed position relative to the V_H_H core. Indeed, there are more predicted trypsin-cleavage sites in the CDR regions (Table S3; Fig. S6) of trypsin-sensitive V_H_Hs (A4.2, A19.2 and A26.8) compared to trypsin-resistant V_H_Hs (A5.1, A20.1 and A24.1). This is not the case for pepsin and chymotrypsin sensitivities (Table S3; Fig. S6).

Importantly, we also observed an increase in *T*_onset_ temperatures for mutants at the physiological conditions representative of the stomach (pH ≅ 2.0 and 37°C) to values significantly above 37°C (*T*_onset_s from 45°C–53°C). This suggests that the mutants should remain fully folded at 37°C in the stomach, hence resisting pepsin degradation (and denaturation) to a higher extent than wild-type V_H_Hs, a statement supported by our *in vitro* pepsin digestion experiments. In contrast to the mutants, 3 wild-type V_H_Hs, for example, have low *T*_onset_ values of 37.8°C (A5.1 and A20.1) and 40.3°C (A26.8) which suggests they would partially unfold in the stomach (pH ≅ 2.0, 37°C), increasing their proteolytic susceptibility. This indeed is the case in an *in vitro* setting as A5.1 and A20.1, V_H_Hs with *T*_onset_ temperatures overlapping the physiological temperature, are completely pepsin sensitive, and A26.8 with a *T*_onset_ slightly above the physiological temperature, although somewhat better than the former two, is barely resistant to pepsin (pepsin resistance: ≈3%). In the corresponding pepsin resistant mutants, acquiring resistance parallels an increase in *T*_onset_. In line with these findings, we observe a strong positive correlation between pepsin resistance and *T*_onset_ (Fig. 8), and depending on the melting curve profile, *T*_onset_s may be better predictors of protein pepsin resistance than *T*_m_s.

Compared to other studies involving *in vitro* V_H_H proteolysis, our mutant V_H_Hs performed remarkably well, withstanding near physiological concentrations of pepsin and chymotrypsin and retaining functionality thereafter. Additionally, half of the mutants were trypsin resistant and for those which were not, identification and removal of their cleavage site(s) should be straightforward, e.g., by MS analysis and site-directed mutagenesis. Balan et al [65] note the human stomach contains pepsin concentrations ranging from 500 µg/mL to 1 mg/mL, while Schmidt et al [66] found the average pepsin concentration in the stomach of piglets to be 155 U/mL. Our pepsin digestion assays were performed at 100 µg/mL concentrations, which correspond to 46 U/mL. The most stable V_H_H mutant produced by Harmsen et al [49] using a DNA shuffling approach showed only 21% residual V_H_H remaining after digestion with 100 µg/mL of pepsin. In contrast, our most stable V_H_H (A4.2m) showed 74% residual V_H_H remaining after digestion, while 4 others had residual pepsin resistance values of 24% or higher. In addition, all 4 disulfide bond mutant V_H_Hs retained binding to TcdA after pepsin treatment, confirming their resistance to the protease and retention of functionality.

We also examined the toxin A neutralizing efficacy of our disulfide bond mutant V_H_Hs. Compared to the wild-type V_H_Hs, the mutants were 3–4 fold weaker with respect to toxin A neutralization in cell-based assays, presumably a reflection in the reduced affinities of 3 of 4 V_H_Hs for the toxin. If a more thorough analysis was performed on individual V_H_Hs, it is possible that clone A4.2m, which showed the same affinity as A4.2 for toxin A, might be a more potent neutralizer due to its higher stability. Under stringent conditions *in vivo*, the lower affinity mutants may actually be more efficacious than the higher affinity wild-type V_H_Hs due to their greater stability, as shown elsewhere [50]. Also, a number of methods are available to increase the affinity of the disulfide-stabilized domains, allowing for the creation of superpotent toxin A neutralizing antibodies capable of withstanding a wide range of harsh conditions.

In conclusion, we have shown that the introduction of a second disulfide bond into the hydrophobic core of a panel of llama V_H_Hs increased thermal stability and GI protease resistance; the approach is both effective and general. The approach does not come without some drawbacks, including, reduced affinity, specificity, and expression yield. However, the mutants outperformed the wild-type V_H_Hs under more stringent physiological conditions, which outweighs the reductions in affinity, as noted above. Whether the mutant V_H_Hs are more efficacious than the wild-type V_H_Hs *in vivo* remains to be determined. Based on our results and those of others, we suggest incorporating the non-canonical disulfide bond between position 54 and 78 at the library construction phase and not after the selection/screening phase to avoid adverse side effects on affinity and specificity seen here and in other studies. Other approaches, such as affinity maturation, could be used to overcome losses in target affinity as a result of disulfide bond incorporation. Our mutant V_H_Hs are ideal building blocks for oral therapeutic agents that must survive the harsh GI tract, and provide promising alternatives to antibiotics. The oral administration of therapeutic proteins is of interest to the pharmaceutical and biotechnological industries [29], [63], [67], [68]. Protein-based oral therapeutics have several conceived advantages over systemic administration: convenience, patience compliance, lower cost, pain-free administration, drug purity, flexibility in production source (i.e., bacterial, plant, etc.), and fewer concerns over immunogenicity. Despite the many advantages of orally administering protein therapeutics, few successes have been realized due to the destabilizing environment of the GI tract. Of the major GI proteases, pepsin is considered the primary cause of antibody degradation [29], [35], [49] and hence a major obstacle facing orally delivered antibody therapeutics. Regarding the mutant V_H_Hs generated in this study, the therapeutic efficacy can be further enhanced by improving their affinity (through selection of affinity maturation display libraries) and by formulation. The affinity maturation libraries could yield V_H_Hs which are hyper-stabilized (e.g., high GI protease resistance) in addition to being of ultra-high affinity, if selection pressures (acid, proteases, heat) are applied during the panning stage [43], [45]. Indeed, the correlation between V_H_H pepsin resistance and *T*_m_ suggests that selection under heat should produce pepsin-resistant V_H_Hs. Given their stability profile, the mutants may be resistant to serum degradation, making them efficacious systemic therapeutics if they are coupled to a half-life extending molecule. Other applications for our stabilized domains include: (i) use as delivery agents for mucosal vaccines [69] or (ii) use as robust affinity purification reagents resistant to acidic and heat elution steps. Furthermore, the recent incorporation of these engineered disulfide bonds into human V_H_ sdAbs not only resulted in increased thermal stability, but also markedly reduced V_H_ aggregation [70], suggesting that the introduced disulfide bond imparts a universal stabilizing effect in all immunoglobulin variable domains.

## Supporting Information

Figure S1 **Alignment and comparison of wild-type and mutant V_H_H amino acid sequences.** Wild-type V_H_H sequences are shown with a single disulfide bond between Cys^23^ and Cys^104^. A second disulfide bond was introduced through mutation of Ala^54^/Gly^54^ and Ile^78^ to Cys^54^ (*) and Cys^78^ in framework region 2 (FR2) and FR3, respectively. Disulfide bonds are shown as black lines. Residues colored in blue illustrate the disulfide bond-linked peptides identified by nanoRPLC-ESI-MS analysis on CNBr and trypsin digested mutant V_H_Hs (Fig. 2). Amino acid numbering and CDR designation is based on the IMGT system (http://imgt.cines.fr/).
(TIF)

Figure S2 **Far-UV CD analysis of V_H_Hs at neutral and acidic pH.** CD scans (210 nm–260 nm) were performed at 25°C on V_H_Hs (50 µg/mL) equilibrated for 2 h in 10 mM sodium phosphate buffer (pH 7.3) or 10 mM sodium phosphate buffer+50 mM HCl (pH 2.0). The spectra represent the mean residue ellipticity of 8 data accumulations collected from 2 independent experiments. Raw data were smoothed using the Jasco software and converted to mean residue ellipticity as described in *Methods*. Red lines: wild-type V_H_H at pH 7.3; blue lines: mutant V_H_H at pH 7.3; green lines: wild-type V_H_H at pH 2.0; orange lines: mutant V_H_H at pH 2.0.
(TIF)

Figure S3 **Near-UV CD analysis of V_H_Hs at neutral and acidic pH.** CD scans (250 nm–340 nm) were performed at 25°C on V_H_Hs (250 µg/mL) equilibrated for 2 h in 10 mM sodium phosphate buffer (pH 7.3) or 10 mM sodium phosphate buffer+50 mM HCl (pH 2.0). The spectra represent the mean residue ellipticity from 8 data accumulations collected from 2 independent experiments. Raw data were smoothed using the Jasco software and converted to mean residue ellipticity as described in *Methods*. Red lines: wild-type V_H_H at pH 7.3; blue lines: mutant V_H_H at pH 7.3; green lines: wild-type V_H_H at pH 2.0; orange lines: mutant V_H_H at pH 2.0.
(TIF)

Figure S4 **V_H_H thermal unfolding curves.** (**A**) Thermal unfolding of wild-type and mutant V_H_Hs (50 µg/mL) at pH 7.3 (10 mM sodium phosphate buffer) and pH 2.0 (10 mM sodium phosphate buffer+50 mM HCl) were followed at 215 nm to identify the thermal unfolding midpoint temperature (*T*_m_). The *T*_m_ was determined for each curve by Boltzmann non-linear curve fitting analysis in GraphPad Prism. The goodness of curve fit (r^2^) ranged from 0.9901–0.9995. In the case of V_H_Hs with few lower baseline data points the *T*_m_ is a minimal estimate (*see* Table 3). Red lines: wild-type V_H_H at pH 7.3; blue lines: mutant V_H_H at pH 7.3; green lines: wild-type V_H_H at pH 2.0; orange lines: mutant V_H_H at pH 2.0. (**B**) Raw thermal unfolding data used to generate the normalized curves in (A).
(TIF)

Figure S5 **V_H_H resistance profiles against trypsin and chymotrypsin.** Wild-type (WT) and mutant (Mut) V_H_Hs were digested with 100 µg/mL of chymotrypsin or trypsin for 1 h at 37°C and separated by SDS-PAGE. Resistance values were calculated as in Fig. 6.
(TIF)

Figure S6 **Correlation between V_H_H protease resistance and the number of theoretical proteolytic cleavage sites.** Linear regression between V_H_H protease resistance and the number of theoretical cleavage sites within the whole V_H_H (“Total sites”) or within the IMGT-defined CDR regions (“CDR sites”). Wild-type and mutant V_H_H protease resistance values were combined for each protease. The number of protease cleavage sites was determined as in Table S3. Linear regression analysis was used to analyze the correlation coefficient (r^2^) and significantly non-zero slope of the line (p) in each graph.
(TIF)

Table S1 **Primers used in this study.**
(PDF)

Table S2 **Onset temperatures (** ***T*** **_onset_s) of wild-type and mutant V_H_Hs.**
(PDF)

Table S3 **Theoretical number of protease cleavable sites located within V_H_Hs.**
(PDF)
