# Supplementary figures and images for: Engineered Single-Domain Antibodies with High Protease Resistance and Thermal Stability
Source: PLoS One. 2011 Nov 30;6(11):e28218. doi: 10.1371/journal.pone.0028218 (PMC3227653; doi:10.1371/journal.pone.0028218)

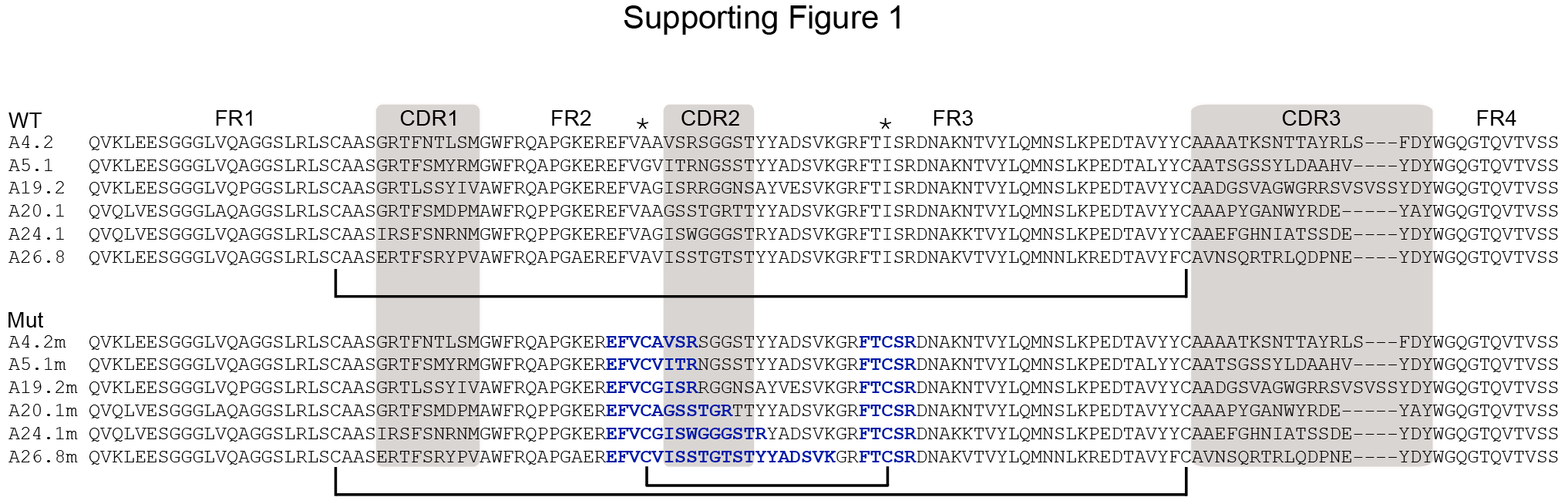

Supplement: Figure S1 — Alignment and comparison of wild-type and mutant VHH amino acid sequences. Wild-type VHH sequences are shown with a single disulfide bond between Cys23 and Cys104. A second disulfide bond was introduced through mutation of Ala54/Gly54 and Ile78 to Cys54 (*) and Cys78 in framework region 2 (FR2) and FR3, respectively. Disulfide bonds are shown as black lines. Residues colored in blue illustrate the disulfide bond-linked peptides identified by nanoRPLC-ESI-MS analysis on CNBr and trypsin digested mutant VHHs (Fig. 2). Amino acid numbering and CDR designation is based on the IMGT system (http://imgt.cines.fr/). (TIF) [file pone.0028218.s001.tif]

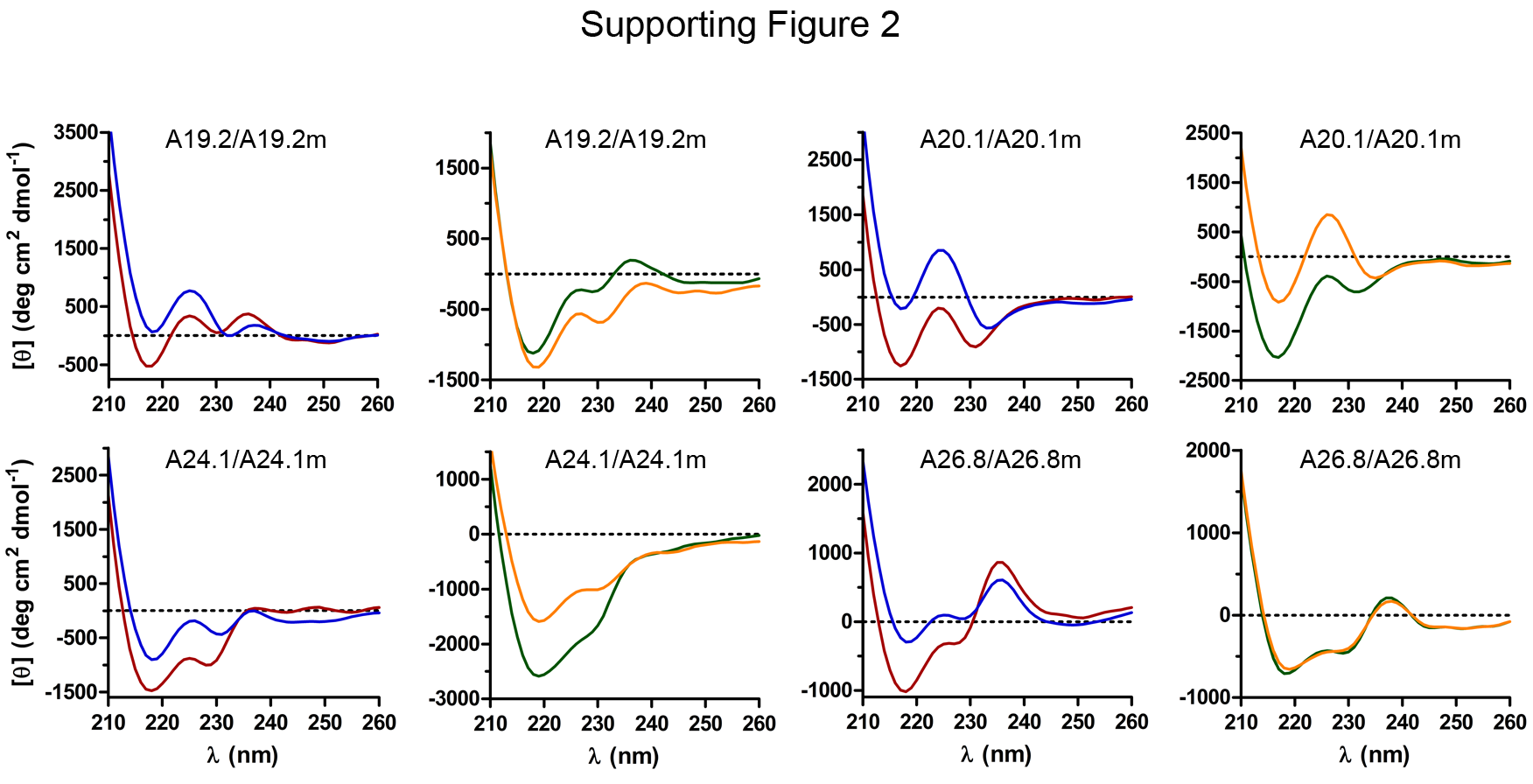

Supplement: Figure S2 — Far-UV CD analysis of VHHs at neutral and acidic pH. CD scans (210 nm–260 nm) were performed at 25°C on VHHs (50 µg/mL) equilibrated for 2 h in 10 mM sodium phosphate buffer (pH 7.3) or 10 mM sodium phosphate buffer+50 mM HCl (pH 2.0). The spectra represent the mean residue ellipticity of 8 data accumulations collected from 2 independent experiments. Raw data were smoothed using the Jasco software and converted to mean residue ellipticity as described in Methods . Red lines: wild-type VHH at pH 7.3; blue lines: mutant VHH at pH 7.3; green lines: wild-type VHH at pH 2.0; orange lines: mutant VHH at pH 2.0. (TIF) [file pone.0028218.s002.tif]

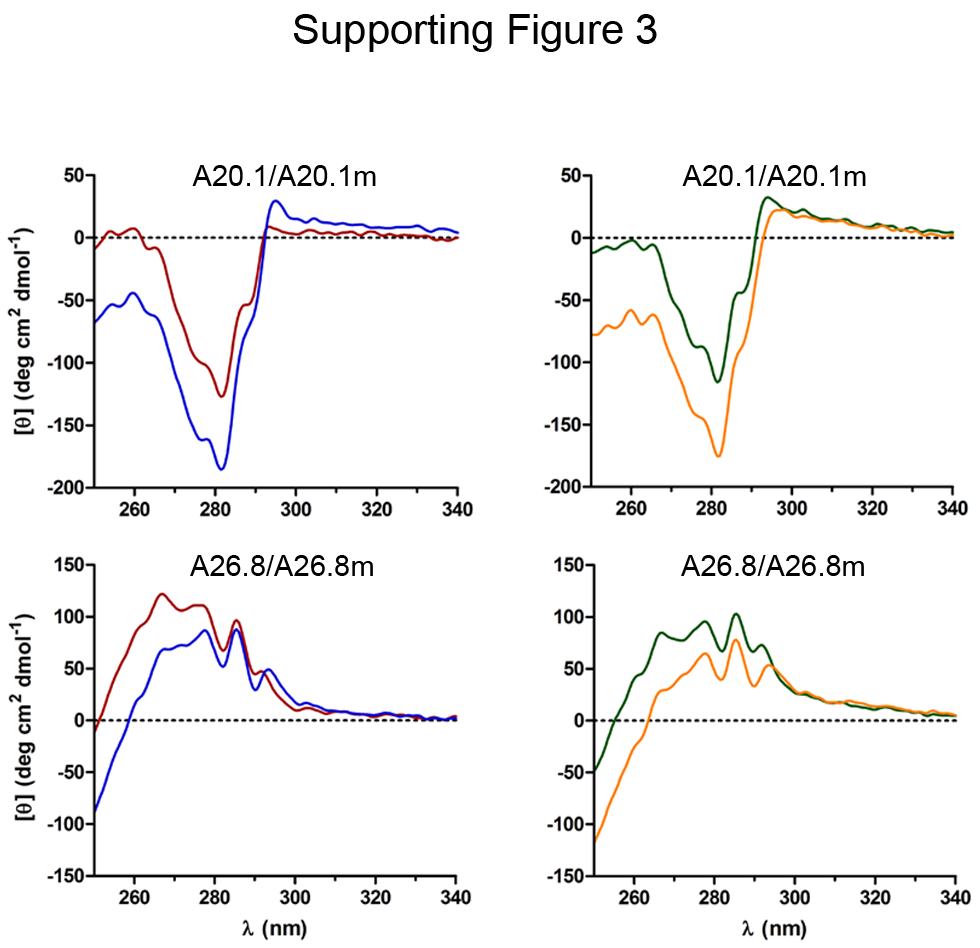

Supplement: Figure S3 — Near-UV CD analysis of VHHs at neutral and acidic pH. CD scans (250 nm–340 nm) were performed at 25°C on VHHs (250 µg/mL) equilibrated for 2 h in 10 mM sodium phosphate buffer (pH 7.3) or 10 mM sodium phosphate buffer+50 mM HCl (pH 2.0). The spectra represent the mean residue ellipticity from 8 data accumulations collected from 2 independent experiments. Raw data were smoothed using the Jasco software and converted to mean residue ellipticity as described in Methods . Red lines: wild-type VHH at pH 7.3; blue lines: mutant VHH at pH 7.3; green lines: wild-type VHH at pH 2.0; orange lines: mutant VHH at pH 2.0. (TIF) [file pone.0028218.s003.tif]

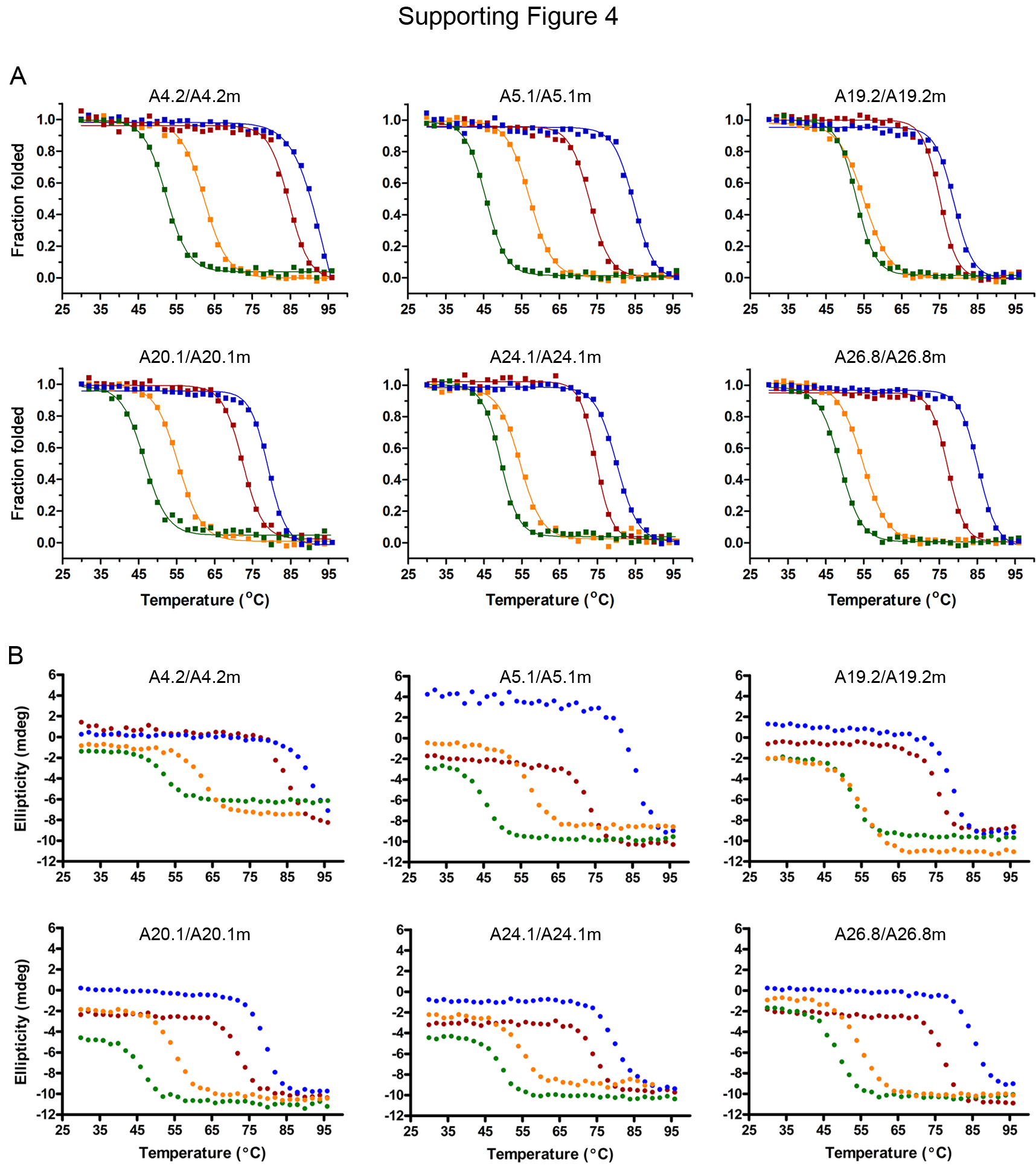

Supplement: Figure S4 — VHH thermal unfolding curves. (A) Thermal unfolding of wild-type and mutant VHHs (50 µg/mL) at pH 7.3 (10 mM sodium phosphate buffer) and pH 2.0 (10 mM sodium phosphate buffer+50 mM HCl) were followed at 215 nm to identify the thermal unfolding midpoint temperature (T m). The T m was determined for each curve by Boltzmann non-linear curve fitting analysis in GraphPad Prism. The goodness of curve fit (r2) ranged from 0.9901–0.9995. In the case of VHHs with few lower baseline data points the T m is a minimal estimate (see Table 3). Red lines: wild-type VHH at pH 7.3; blue lines: mutant VHH at pH 7.3; green lines: wild-type VHH at pH 2.0; orange lines: mutant VHH at pH 2.0. (B) Raw thermal unfolding data used to generate the normalized curves in (A). (TIF) [file pone.0028218.s004.tif]

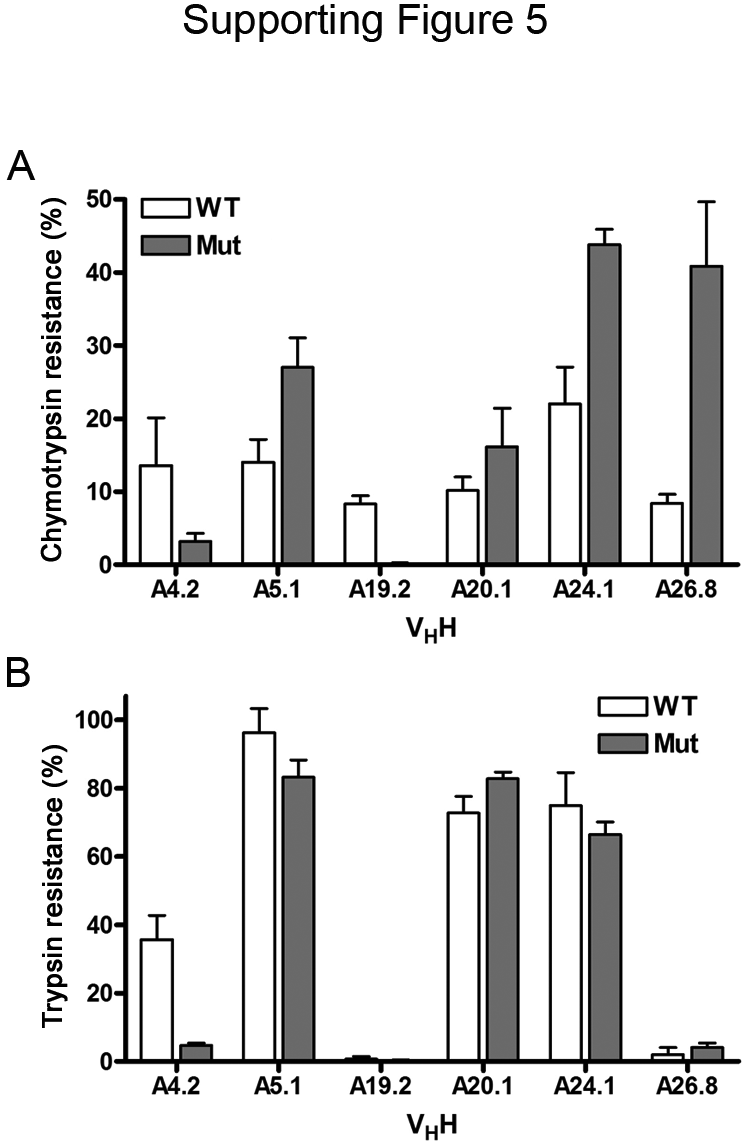

Supplement: Figure S5 — VHH resistance profiles against trypsin and chymotrypsin. Wild-type (WT) and mutant (Mut) VHHs were digested with 100 µg/mL of chymotrypsin or trypsin for 1 h at 37°C and separated by SDS-PAGE. Resistance values were calculated as in Fig. 6. (TIF) [file pone.0028218.s005.tif]

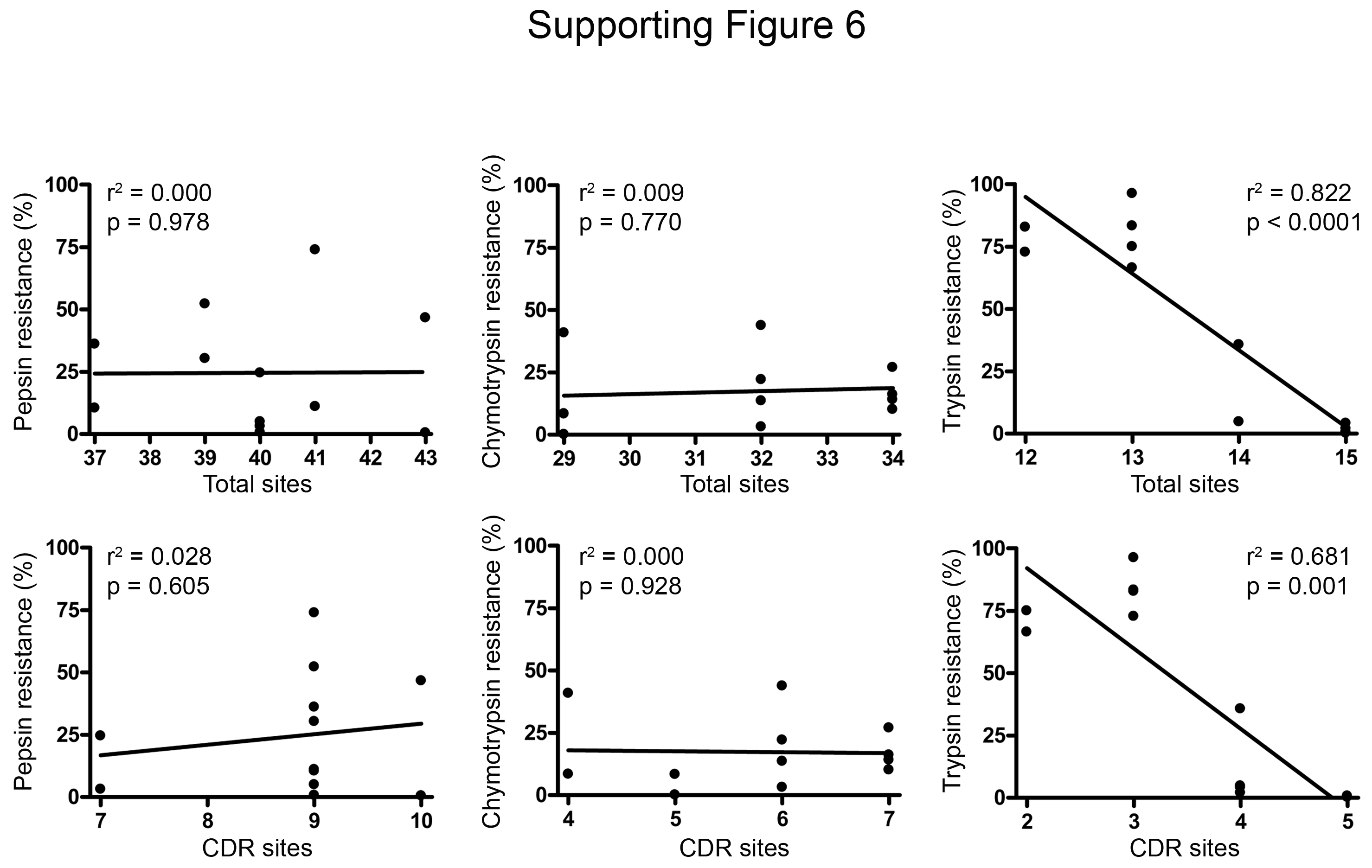

Supplement: Figure S6 — Correlation between VHH protease resistance and the number of theoretical proteolytic cleavage sites. Linear regression between VHH protease resistance and the number of theoretical cleavage sites within the whole VHH (“Total sites”) or within the IMGT-defined CDR regions (“CDR sites”). Wild-type and mutant VHH protease resistance values were combined for each protease. The number of protease cleavage sites was determined as in Table S3. Linear regression analysis was used to analyze the correlation coefficient (r2) and significantly non-zero slope of the line (p) in each graph. (TIF) [file pone.0028218.s006.tif]
