# Supplementary material for: Engineered Single-Domain Antibodies with High Protease Resistance and Thermal Stability
Source: PLoS One. 2011 Nov 30;6(11):e28218. doi: 10.1371/journal.pone.0028218 (PMC3227653; doi:10.1371/journal.pone.0028218)
Supplement: Table S1 — Primers used in this study. (PDF) [file pone.0028218.s007.pdf]

Table S1: Primers used in this study.

**Primers**	**Sequence (5' → 3')**	**Purpose**
M13FP	GTAAAACGACGGCCAGT	Screening
M13RP	CAGGAAACAGCTATGAC	Screening
BbsI-VHH[1]	TATGAAGACACCAGGCCCAGGTAAAGCTGGAGGAGTCT	Constructing mutants
BamHI-VHH[1]	TTGTTCGGATCCTGAGGAGACGGTGACCTG	Constructing mutants
A4.2mR-Cys	AGTCTGCATAGTATGTGCTACCACCACTCCGGCTAACAGCGCAAACAAACTC	Constructing A4.2m
A4.2mF-Cys	TAGCACATACTATGCAGACTCCGTGAAGGGCCGATTCACCTGCTCCAGAGAC	Constructing A4.2m/A5.1m
A5.1mR-Cys	AGTCTGCATAGTATGTGCTACTACCATTCCGGGTAATAACGCATACAAACTC	Constructing A5.1m
A19.2mR-Cys	ACTCTACATAGGCACTATTACCACCACGCCGGCTAATACCGCATACAAACTC	Constructing A19.2m
A19.2mF-Cys	TAATAGTGCCTATGTAGAGTCCGTGAAGGGCCGATTCACCTGCTCCAGAGAC	Constructing A19.2m
A20.1mSfiI-F	ACCGTTGCGCAGGCCCAGCCGGCCATGGCCCAGGTACAGC	Constructing A20.1m/A24.1m
A20.1mR-Cys	TGTCTGCATAGTATGTGGTCCGCCCCGTAGAACTCCCCGCGCATACAAACTC	Constructing A20.1m
A20.1mF-Cys	GACCACATACTATGCAGACAGCGTGAAGGGCCGATTCACCTGCTCCAGAGAC	Constructing A20.1m
A20.1mSfiI-R	GTTCGGATCCCTGGCCGGCCTGGCCTGAGGAGACGGTGACC	Constructing A20.1m/A24.1m
A24.1mR-Cys	AGTCTGCATAGCGTGTGCTACCTCCACCCCAGCTAATACCGCATACAAACTC	Constructing A24.1m
A24.1mF-Cys	TAGCACACGCTATGCAGACTCCGTGAAGGGCCGATTCACCTGCTCCAGAGAC	Constructing A24.1m
A26.8mR-Cys	AGTCTGCATAGTATGTGCTCGTACCAGTCGAGCTAATAACGCATACAAACTC	Constructing A26.8m
A26.8mF-Cys	GAGCACATACTATGCAGACTCGGTGAAGGGCCGGTTCACCTGCTCCAGAGAC	Constructing A26.8m

[1] Reverse and forward primers for construction of A4.2m, A5.1m, A19.2m, and A26.8m.
