# Supplementary material for: Engineered Single-Domain Antibodies with High Protease Resistance and Thermal Stability
Source: PLoS One. 2011 Nov 30;6(11):e28218. doi: 10.1371/journal.pone.0028218 (PMC3227653; doi:10.1371/journal.pone.0028218)
Supplement: Table S2 — Onset temperatures ( T onsets) of wild-type and mutant VHHs. (PDF) [file pone.0028218.s008.pdf]

Table S2: Onset temperatures (T_{onset}s) of wild-type and mutant V_HHs.

**V_HH**	**T_{onset} pH 7.3 (°C)**		**T_{onset} pH 2.0 (°C)**	
	**Wild-type**	**Mutant**	**Wild-type**	**Mutant**
A4.2/ A4.2m	76.5	80.0	43.7	53.1
A5.1/ A5.1m	65.2	76.6	37.8	48.4
A19.2/ A19.2m	68.3	71.4	45.3	45.0
A20.1/ A20.1m	64.6	72.0	37.8	46.3
A24.1/ A24.1m	68.2	71.7	42.2	46.0
A26.8/ A26.8m	70.7	77.8	40.3	45.2

T_{onset} is defined as the temperature at which 5% of the V_HH is unfolded.
