# Supplementary material for: Engineered Single-Domain Antibodies with High Protease Resistance and Thermal Stability
Source: PLoS One. 2011 Nov 30;6(11):e28218. doi: 10.1371/journal.pone.0028218 (PMC3227653; doi:10.1371/journal.pone.0028218)
Supplement: Table S3 — Theoretical number of protease cleavable sites located within VHHs. (PDF) [file pone.0028218.s009.pdf]

**Table S3:** Theoretical number of protease cleavable sites located within $V_H$Hs[a].

| $V_H$H | Pepsin[b] | Chymotrypsin[c] | Trypsin |
|---|---|---|---|
| A4.2/ A4.2m | 41-3-0-6-(9) | 32-2-0-4-(6) | 14-1-1-2-(4) |
| A5.1/ A5.1m | 43-3-0-7-(10) | 34-2-0-5-(7) | 13-2-1-0-(3) |
| A19.2/ A19.2m | 39-3-0-6-(9) | 29-2-0-3-(5) | 15-1-2-2-(5) |
| A20.1/ A20.1m | 40-1-0-8-(9) | 34-2-0-5-(7) | 12-1-1-1-(3) |
| A24.1/ A24.1m | 37-1-2-6-(9) | 32-1-1-4-(6) | 13-2-0-0-(2) |
| A26.8/ A26.8m | 40-2-0-5-(7) | 29-1-0-3-(4) | 15-2-0-2-(4) |

Table notation: $V_H$H Total-CDR1-CDR2-CDR3-(CDR sum)

[a] Using the Expasy PeptideCutter (http://www.expasy.ch/tools/peptidecutter/) tool.

[b] Selected for “pH > 1.3”.

[c] Selected for “low specificity”.
